# The *Escherichia coli* effector EspJ blocks Src kinase activity via amidation and ADP ribosylation

**DOI:** 10.1038/ncomms6887

**Published:** 2014-12-19

**Authors:** Joanna C. Young, Abigail Clements, Alexander E. Lang, James A. Garnett, Diana Munera, Ana Arbeloa, Jaclyn Pearson, Elizabeth L. Hartland, Stephen J. Matthews, Aurelie Mousnier, David J. Barry, Michael Way, Andreas Schlosser, Klaus Aktories, Gad Frankel

**Affiliations:** 1 MRC Centre for Molecular Bacteriology and Infection, Imperial College, SW7 2AZ London, UK; 2 Institute of Experimental and Clinical Pharmacology and Toxicology, University of Freiburg, D-79104 Freiburg, Germany; 3 Centre for Structural Biology, Imperial College, SW7 2AZ London, UK; 4 Department of Microbiology and Immunology, University of Melbourne, at the Peter Doherty Institute for Infection and Immunity, Melbourne Victoria 3010, Australia; 5 Cell Motility Laboratory, Cancer Research UK, London Research Institute, 44 Lincoln’s Inn Fields, London WC2A 3LY, UK; 6 Rudolf Virchow Center for Experimental Biomedicine, University of Wuerzburg, 97080 Würzburg, Germany; 7 Centre for Biological Signalling Studies (BIOSS), University of Freiburg, D-79104 Freiburg, Germany

## Abstract

The hallmark of enteropathogenic *Escherichia coli* (EPEC) infection is the formation of actin-rich pedestal-like structures, which are generated following phosphorylation of the bacterial effector Tir by cellular Src and Abl family tyrosine kinases. This leads to recruitment of the Nck–WIP–N-WASP complex that triggers Arp2/3-dependent actin polymerization in the host cell. The same phosphorylation-mediated signalling network is also assembled downstream of the Vaccinia virus protein A36 and the phagocytic Fc-gamma receptor FcγRIIa. Here we report that the EPEC type-III secretion system effector EspJ inhibits autophosphorylation of Src and phosphorylation of the Src substrates Tir and FcγRIIa. Consistent with this, EspJ inhibits actin polymerization downstream of EPEC, Vaccinia virus and opsonized red blood cells. We identify EspJ as a unique adenosine diphosphate (ADP) ribosyltransferase that directly inhibits Src kinase by simultaneous amidation and ADP ribosylation of the conserved kinase-domain residue, Src E310, resulting in glutamine-ADP ribose.

## Abstract (web summary)

Non-receptor tyrosine kinases such as Src play fundamental roles in host–pathogen interactions and phagocytosis. Here, Young *et al*. show that an enteropathogenic *Escherichia coli* (EPEC) protein, EspJ, inhibits Src activity by simultaneous amidation and ADP ribosylation of a conserved residue on the kinase.

## Graphical abstract

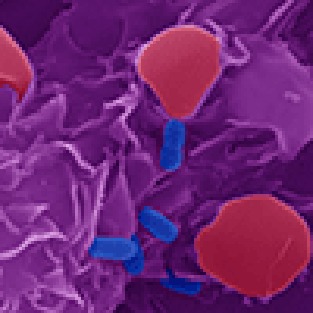

The tyrosine Src family kinases (SFKs) play a fundamental role in a wide variety of cellular processes including morphogenesis and proliferation^1^, phagocytosis^2^ and host–pathogen interactions^3^,^4^. Furthermore, SFKs are overexpressed and/or aberrantly activated in a wide variety of cancers^5^. In humans, SFKs comprise eight members, with Src, Fyn and Yes being ubiquitously expressed^6^. SFKs consist of an N-terminal myristoylation/palmitoylation site, SH3 and SH2 protein-interaction domains and a C-terminal kinase domain (SH1). In their inactive state, SFKs assume an autoinhibited conformation that is mediated by intramolecular interactions^7^ (Supplementary Fig. 1). Interaction between the SH2 domain and a C-terminal tyrosine Y527, when it is phosphorylated by Csk, promotes the autoinhibited conformation^8^,^9^, while removal of Y527 results in a constitutively activated kinase^10^. Dephosphorylation of Y527 as well as binding of ligands to the SH2 or SH3 domains alleviates the autoinhibitory state of Src, leading to autophosphorylation of Y416 and maximal kinase activity^10^,^11^. Upon binding of immunoglobulin (Ig)G-coated particles to FcγRs, active SFK phosphorylate the immunoglobulin tyrosine activation motif of FcγR^2^, which in turn initiates actin-driven opsono-phagocytosis downstream of Cdc42, Rac1, Nck and N-WASP^12^,^13^. We previously reported that opsono-phagocytosis via FcγRIIa could be inhibited by the enteropathogenic *Escherichia coli* (EPEC) and enterohaemorrhagic *E. coli* (EHEC) effector EspJ through an unknown mechanism^14^. Here we show that EspJ inhibits opsono-phagocytosis through inactivation of Src, disrupting phosphorylation of the FcγRIIa. EspJ inhibits Src activity by a unique post-translational modification mechanism involving amidation and adenosine diphosphate (ADP) ribosylation of a key kinase-domain residue, which is conserved across the protein kinase superfamily.

## Results

### EspJ resembles ADP ribosyltransferases

**Figure 1:**
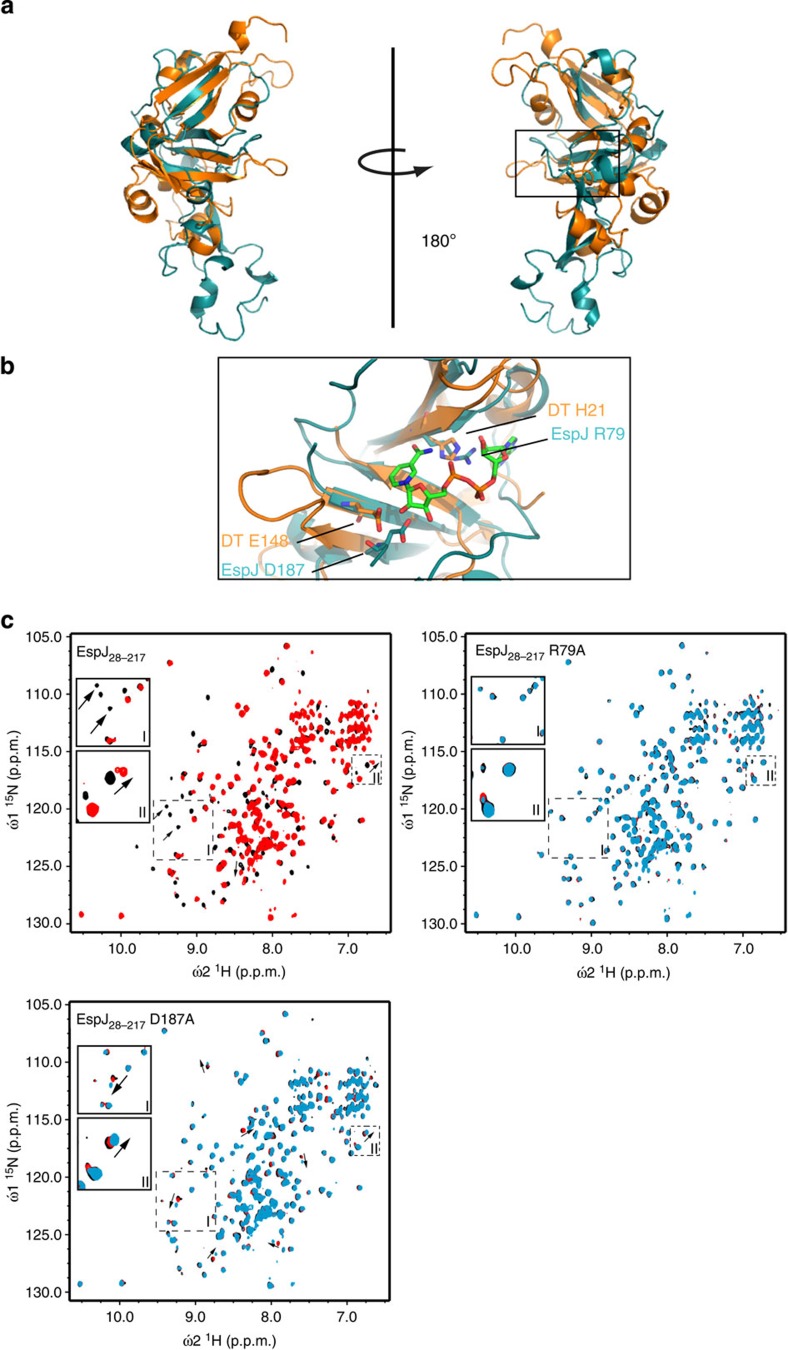
EspJ ART domain residues are required for NAD^+^ binding. (**a**) Overlay of a model of EspJ (cyan) and the canonical ART diphtheria toxin (DT) (orange). The NAD^**+**^-binding region is highlighted by a black box and magnified in **b** (NAD^+^ C shown in green, O in red, N in blue and P in orange). The side chains of R79, D187 (EspJ) and H21, E148 (diphtheria toxin) are shown (with O in red and N in blue). (**c**) Two-dimensional ^1^H–^15^N HSQC NMR spectra of recombinant ^15^N-labelled EspJ_28-217_, EspJ_28-217_ R79A and EspJ_28-217_ D187A (black) in the presence of 1 (red) or 10-fold (cyan) molar equivalents of NAD^**+**^. Arrows highlight examples of spectral changes upon addition of NAD^**+**^. Enlarged boxes show spectral changes in the EspJ_28-217_ spectra and equivalent peaks in EspJ_28-217_ R79A and EspJ_28-217_ D187A spectra.

The Phyre protein fold recognition server^15^ indicated that extensive structural homology exists between EspJ and the ADP-ribosyltransferase (ART) domain of the *Pseudomonas syringae* effector AvrPphF-ORF2 (ref. 16) (*E*-value=1.3 × 10^−8^), including the β-sheet fold characteristic of ARTs (Fig. 1a,b; Supplementary Fig. 2). ARTs mediate transfer of ADP ribose from nicotinamide adenine dinucleotide (NAD^+^) onto target proteins, modulating their interactions and subsequent signalling^17^. To explore whether EspJ can bind NAD^+^, we recorded ^1^H–^15^N two-dimensional heteronuclear single quantum coherence (HSQC) nuclear magnetic resonance (NMR) spectra of recombinant ^15^N-labelled EspJ_28-217_ (lacking the N-terminal secretion signal) in the presence and absence of NAD^+^. The addition of NAD^+^ caused substantial chemical shift perturbations for several resonances, consistent with a significant interaction with EspJ (Fig. 1c).

To characterize this interaction further, we mutated R79 and D187 in EspJ, which would contribute directly to NAD^+^ binding and catalytic activity based on comparisons with AvrPphF-ORF2 and the canonical ART diphtheria toxin^16^,^18^ (Fig. 1a,b; Supplementary Fig. 2). While the positions of some ^1^H–^15^N HSQC peaks for EspJ_28-217_R79A and EspJ_28-217_D187A were altered relative to the EspJ_28-217_ spectra, the excellent spectra dispersion indicates that the mutations have not affected the overall protein structure. Upon titration with NAD^+^ no significant chemical shift perturbations were observed for EspJR79A. When NAD^+^ was present at a 10-fold molar equivalent some altered chemical shifts were observed for EspJ28-217 D187A, consistent with D187 contributing to NAD binding, but not being essential for recognition (Fig. 1c).

### NAD binding is necessary for the biological activity of EspJ

**Figure 2:**
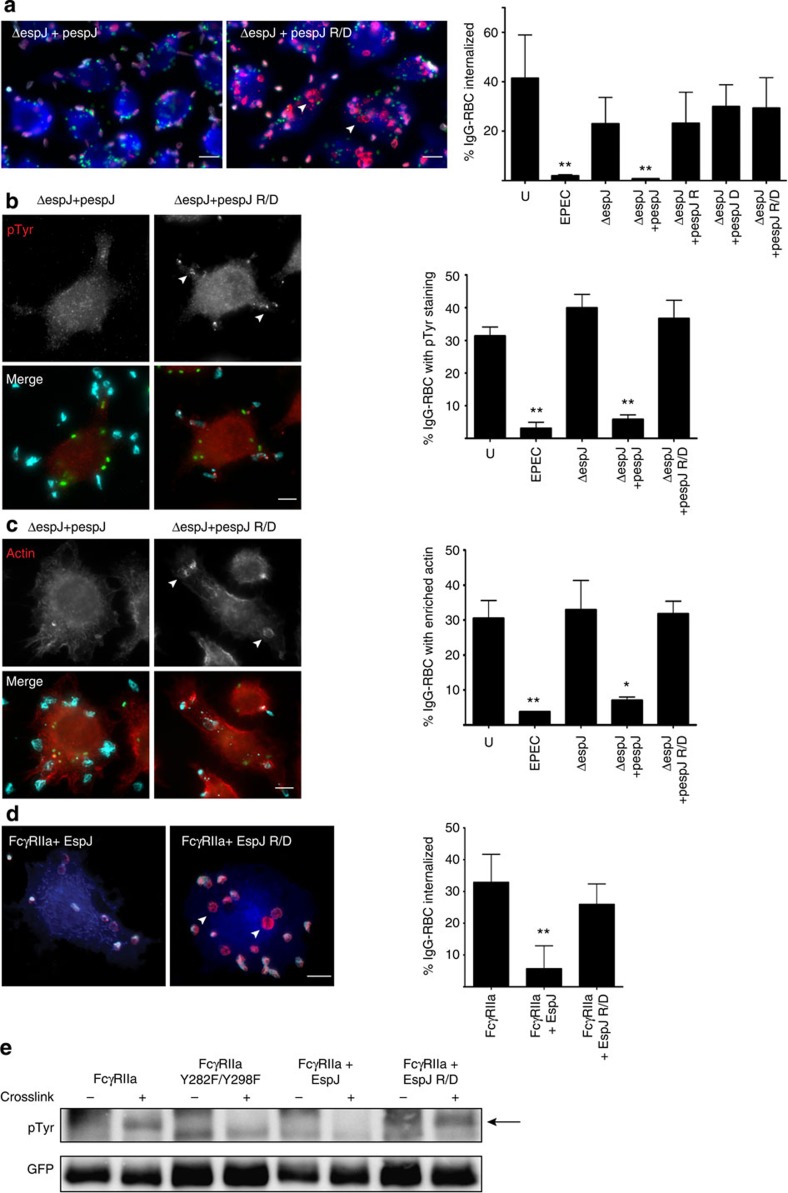
EspJ inhibits FcγRIIa tyrosine phosphorylation and downstream actin polymerization. (**a**–**c**) Macrophages were infected with GFP-EPEC, EPECΔe*spJ* or EPECΔe*spJ* expressing plasmid-encoded EspJ (pespJ), EspJ-R, EspJ-D or EspJ-R/D and then were challenged with IgG-opsonized RBC. Internalized RBCs (**a**) (total RBC are shown in red, external RBC in both red and cyan and actin in blue), tyrosine phosphorylation (**b**) and actin polymerization (**c**) (arrowheads) were visualized. Scale bars, 10 μm. Only EPEC and EPECΔe*spJ* expressing EspJ reduced RBC phagocytosis, tyrosine phosphorylation and actin polymerization. (**d**) Cos-7 cells co-expressing FcγRIIa and myc-tagged (blue) EspJ, or EspJ-R/D were challenged with IgG-opsonized RBC and the percentage of internalized RBC quantified (arrowheads). Scale bars, 10 μm. Again, only EspJ could inhibit RBC phagocytosis. Results are the mean±s.d. of three independent experiments in which 100 (**a**) or 50 (**b**–**d**) cells were analysed. Data sets were analysed using one-way analysis of variance (GraphPad Prism v6.0). A significant result is defined as *P*<0.05 (shown as * and *P*<0.01 shown as **) as compared with uninfected or untransfected controls. (**e**) Cos-7 cells co-expressing GFP-tagged FcγRIIa or FcγRIIa Y282F/Y298F with EspJ, EspJ-R/D or an empty vector were treated with anti-FcγR IV.3 antibody with or without secondary antibody crosslinking. The FcγRIIa was immunoprecipitated and analysed by immunoblotting with anti-pTyr and anti-GFP antibodies, which shows phosphorylation of wild-type FcγRIIa in the control and EspJ-R/D-expressing cells (arrow), but not in cells expressing EspJ or FcγRIIa Y282F/Y298F. Similar results were obtained in three independent experiments. Representative immunoblots are shown. Full immunoblots are shown in Supplementary Fig. 3.

To identify the cellular targets of EspJ, we first examined whether the EspJ-R (R79A), -D (D187A) and -R/D (R79A/D187A) mutants could inhibit phagocytosis of opsonized red blood cells (RBCs). J774A.1 macrophages were infected with EPEC and subsequently challenged with IgG-coated RBC (IgG-RBC). Infection with EPEC resulted in inhibition of phagocytosis of IgG-RBC to <2% compared with ~40% in uninfected cells (Fig. 2a). In contrast, there was ~23% RBC internalization in cells infected with EPECΔ*espJ*. The level of phagocytosis inhibition was restored by complementation with plasmid-encoded EspJ (pespJ), but not EspJ-R, -D or -R/D mutants (Fig. 2a). J774A.1 cells infected with EPEC and challenged with IgG-RBC also revealed that only EPEC or EPECΔ*espJ*-expressing EspJ, but not EspJ-R/D, significantly reduced the level of pTyr and actin accumulation at RBC attachment sites (Fig. 2b,c). In addition, co-expression of EspJ and FcγRIIa in Cos-7 cells, outside the context of EPEC infection, demonstrated that EspJ, but not EspJ-R/D, significantly reduced RBC internalization (Fig. 2d). Immunoblot analysis of FcγRIIa, crosslinked with anti-FcγRIIa antibodies, demonstrated that expression of EspJ, but not EspJ-R/D, reduced phosphorylation of FcγRIIa to that of its non-phosphorylatable Y282F/Y298F mutant (Fig. 2e; Supplementary Fig. 3). This suggests that EspJ suppresses phagocytosis by inhibiting SFK-mediated phosphorylation of FcγRIIa.

### EspJ blocks the kinase activity of Src

**Figure 3:**
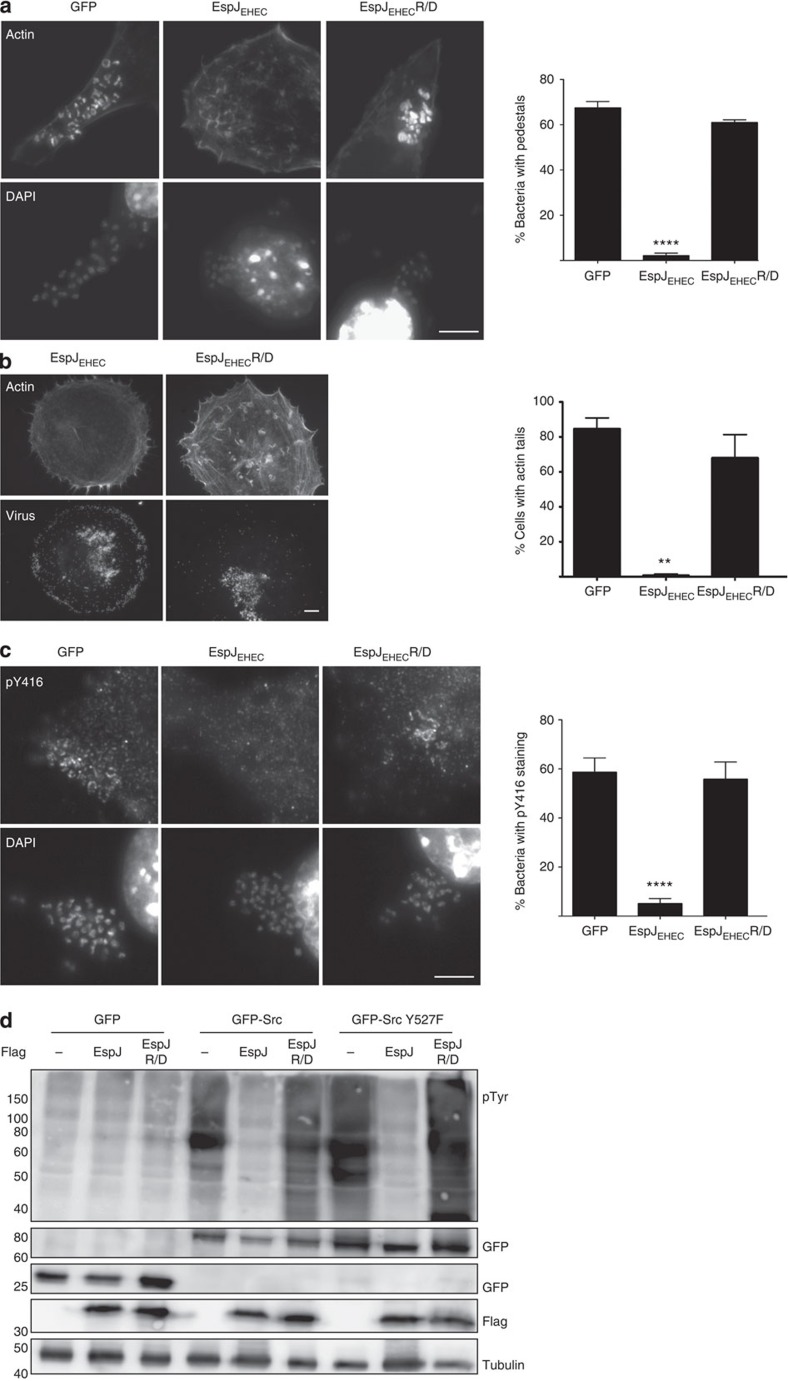
EspJ inhibits EPEC- and Vaccinia virus-induced actin polymerization and recruitment of active Src to sites of bacterial attachment. Cultured cells expressing Flag-tagged GFP, EspJ or, EspJ-R/D were infected with EPEC (**a**) or Vaccinia virus (**b**) and were quantified for actin polymerization associated with attached EPEC (DAPI stained) or Vaccinia virions (RFP-tagged A3 viral core protein). EspJ, but not EspJ-R/D, inhibited actin polymerization under both EPEC and Vaccinia virions. (**c**) Cells expressing Flag-tagged GFP, EspJ, or EspJ-R/D, were infected with EPEC and stained with anti-pY416, to detect active SFKs, and 4',6-diamidino-2-phenylindole (DAPI). EspJ, but not EspJ-R/D, inhibited accumulation of pY416 under adherent EPEC. Fifty cells were analysed in each of three independent experiments. Data sets were analysed using one-way analysis of variance (GraphPad Prism v6.0). A significant result is defined as *P*<0.05 (*P*<0.01 shown as ** and *P*<0.0001 shown as ****) as compared with control infections. Scale bars, 10 μm (**a**,**c**) or 20 μm (**b**). (**d**) Swiss 3T3 cells co-expressing GFP, Src-GFP or Src Y527F-GFP with Flag-tagged EspJ, EspJ-R/D or an empty vector were analysed by anti-pTyr immunoblot. EspJ inhibited general protein tyrosine phosphorylation. Similar results were obtained in three independent experiments. Representative immunoblots are shown. Full immunoblots are shown in Supplementary Fig. 7.

To determine whether other Src-dependent phosphorylation events were also inhibited, we examined the impact of EspJ on EPEC and Vaccinia virus-induced actin polymerization. The ability of EPEC and Vaccinia virus to stimulate actin polymerization is dependent on Src- and Abl-mediated phosphorylation of Tir and A36, respectively^3^,^4^,^19^,^20^. Phosphorylation of Tir and A36 results in recruitment of a signalling network consisting of Nck, WIP and N-WASP, which is required for Arp2/3-dependent actin polymerization^9^,^21^. We found that ectopically expressed EspJ, but not EspJ-R/D, inhibited actin polymerization induced by EPEC and Vaccinia virus (Fig. 3a,b). In contrast, EspJ had no impact on the phosphorylation-independent actin polymerization ability of EHEC O157:H7 (which naturally expresses the type-III secretion system effector TccP/EspF_U_) or EPEC transformed with a plasmid encoding TccP/EspF_U_ (Supplementary Fig. 4a,b). This is not surprising as TccP/EspF_U_-driven actin polymerization circumvents the requirement of Tir tyrosine phosphorylation by directly activating N-WASP independently of Nck^9^,^22^.

**Figure 4:**
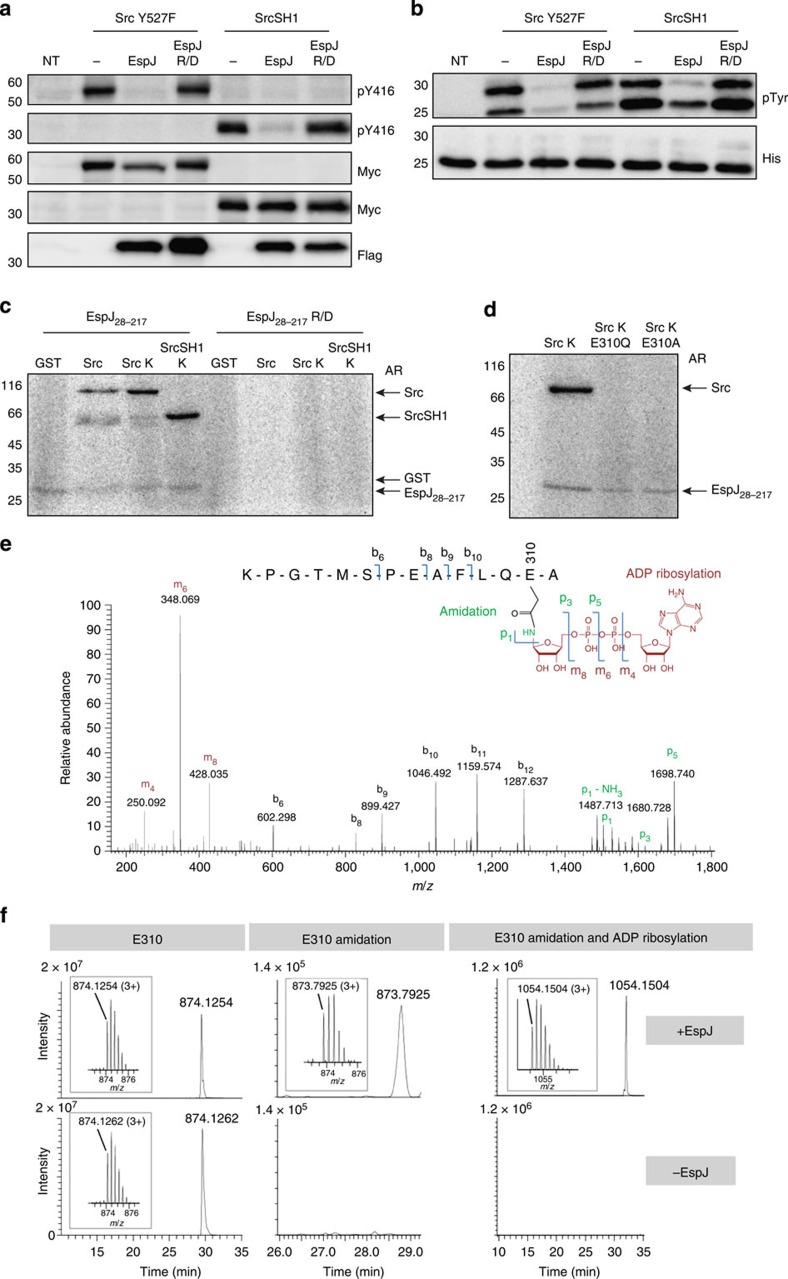
EspJ inhibits Src kinase activity by amidation and ADP ribosylation of Src E310. (**a**) Swiss 3T3 cells co-expressing myc-tagged SrcY527F or SrcSH1 with Flag-tagged EspJ, or EspJ-R/D were analysed by anti-pY416, anti-Myc and anti-Flag antibodies. EspJ inhibited autophosphorylation (pY416) of both SrcY527F and SrcSH1. (**b**) Myc-tagged SrcY527F or SrcSH1 were immunoprecipitated from lysates of cells expressing EspJ or EspJ-R/D and incubated with His-tagged TirC_EPEC_. Analysis of TirC_EPEC_ by immunobloting with anti-pTyr and anti-His antibodies indicated that SrcY527F and SrcSH1 were inactive when immunoprecipitated from cells co-expressing EspJ. Similar results were obtained in three independent experiments. Representative immunoblots are shown. The full immunoblots are shown in Supplementary Fig. 8. (**c**) Recombinant EspJ and EspJ EspJ-R/D were incubated with GST, GST-Src, GST-Src K295M (Src K) or GST-SrcSH1 K295M (SrcSH1 K) and ^32^P-labelled NAD^+^. Autoradiograph (AR) showing ^32^P-labelled Src and SrcSH1 in the presence of EspJ, but not EspJ-R/D. Corresponding commassie-stained PAGE gels are shown in Supplementary Fig. 9. Similar results were obtained in three independent experiments. (**d**) Recombinant EspJ was incubated with GST-Src K295M (Src K), GST-SrcK-E310Q or GST-SrcK-E310A and ^32^P-labelled NAD^+^. Autoradiograph showing ^32^P-labelling of only Src K. Similar results were obtained in two independent experiments. (**e**) HCD MS/MS spectrum of the precursor 1023.4071 (2+) corresponding to the amidated and ADP-ribosylated peptide with the sequence 298-KPGTMSPEAFLQEA-311 generated by digest of EspJ-incubated Src-K295M with elastase. Collision-induced fragmentation is observed at the backbone (b ion series) as well as in the ADP-ribose moiety (m and p ions). All p ions are shifted by −0.984 Da indicating the amidation (O to NH exchange) at the side chain of E310. (**f**) Src-K295M was incubated with or without addition of EspJ. Proteins were separated by SDS–PAGE, digested and analysed by nanoLC-MS/MS. Extracted ion chromatograms for the differentially modified tryptic peptide VAIMTLKPGTMSPEAFLQEAQVMK are shown: the unmodified sequence (E310), the amidated form of this peptide (E310 amidation) and the amidated and ADP-ribosylated form (E310 amidation and ADP ribosylation). The unmodified form of the tryptic peptide is detected in the presence and in the absence of EspJ, whereas the modified forms are only detectable when Src-K295M has been incubated with EspJ. Results were consistent in a repeat experiment using SrcSH1.

While ectopic expression of EspJ did not interfere with Tir translocation, a marked reduction in Tir phosphotyrosine staining and Nck recruitment at bacterial attachment sites was observed in cells ectopically expressing EspJ, but not EspJ-R/D (Supplementary Fig. 5a–c). Moreover, EspJ, but not EspJ-R/D, decreased the level of active SFKs (pY416) beneath adherent EPEC, although green fluorescent protein (GFP)-Src was recruited to bacterial attachment sites (Fig. 3c; Supplementary Fig. 6a). Consistent with this, we found that EspJ inhibited EPEC-induced actin polymerization even in the presence of constitutively active SrcY527F (Supplementary Fig. 6b). Furthermore, immunoblot analysis demonstrated that EspJ inhibits the marked increase in levels of tyrosine-phosphorylated cellular proteins seen following expression of Src or constitutively active SrcY527F, as well as autophosphorylation of the kinase on Y416 (Figs 3d and 4a; Supplementary Fig. 7). This inhibition was absent in cells expressing EspJ-R/D (Figs 3d and 4a).

To test whether the inhibition is due to direct inactivation of the kinase itself, we measured the kinase activity of SrcY527F immunoprecipitated from cells co-expressing EspJ or EspJ-R/D (Fig. 4b; Supplementary Fig. 8). We found that SrcY527F immunoprecipitated from cells expressing EspJ-R/D, but not EspJ, readily phosphorylated the C terminus of Tir_EPEC_ (TirC) forming a doublet on α-pTyr immunoblot, suggesting that Tir is phosphorylated on multiple tyrosines by Src (Fig. 4b). Furthermore, EspJ also inhibited autophosphorylation of the kinase domain in Src (SH1), as well as phosphorylation of Tir *in vitro* (Fig. 4a,b). Interestingly, one of the phosphorylated Tir bands was still observed in the presence of SrcSH1, likely due to incomplete inhibition by EspJ. SrcSH1 immunoprecipitated from cells expressing EspJ-R/D was, as expected, fully active (Fig. 4b). Taken together, these results demonstrate that EspJ permanently inactivates the activity of Src, in a NAD^+^-binding-dependent manner, by directly targeting its SH1 kinase domain.

### EspJ amidates and ADP-ribosylates Src

We next investigated whether the inactivation of Src was through ADP ribosylation by performing an *in vitro* ADP-ribosylation assay on purified glutatione S-transferase (GST)-tagged Src, Src-K295M (kinase inactive) and SrcSH1-K295M in the absence and presence of EspJ or EspJ-R/D. Using radiolabelled NAD^+^ revealed that EspJ, but not EspJ-R/D, could ADP-ribosylate the three Src proteins (Fig. 4c; Supplementary Fig. 9).

The site of Src ADP-ribosylation was then determined by mass spectrometry (MS). *In vitro* ADP-ribosylated Src-K295M was separated by SDS–polyacrylamide gel electrophoresis (PAGE), and the Coomassie-stained protein band was excised and digested with either trypsin, thermolysin or elastase. All digests were analysed by nanoliquid chromatography (LC)-MS/MS using higher-energy C-trap dissociation (HCD), as well as electron-transfer dissociation (ETD) fragmentation. HCD spectra of ADP-ribosylated peptides were identified by filtering the spectra for the presence of ADP-ribosyl-specific marker fragments (*m*/*z* 250.09, 348.07 and 428.04)^23^. The spectrum shown in Fig. 4e shows typical ADP-ribosyl-specific marker fragments (for example, m_4_, m_6_ and m_8_) as well as a series of b ions (b_8_–b_12_) that enabled the identification of the peptide, as well as the localization of the ADP ribosylation to E310. Unexpectedly, in addition to the ADP ribosylation, E310 was also amidated, so that E310 was converted to Q310. This finding was confirmed by searching all fragment ion spectra against a custom database containing the sequences of both Src-K295M and Src-K295M/E310Q. Whereas no ADP-ribosylated peptide was mapped to the sequence of Src-K295M, 27 ADP-ribosylated peptides (all containing Q310) were mapped to the sequence of Src-K295M/E310Q (Supplementary Table 4). ADP ribosylation of E310 was completely abolished when E310 was mutated to either A or Q (Fig. 4d). Additional evidence for the simultaneous modification of E310 of Src-K295M by amidation and ADP ribosylation came from a comparison of the tryptic digests of EspJ-treated and untreated Src-K295M (Fig. 4f). As expected, the tryptic peptide modified by amidation and ADP ribosylation was only detectable in the EspJ-treated sample. However, in addition we were able to identify the same tryptic peptide modified by amidation (E310Q) only, specifically in the EspJ-treated sample (Supplementary Fig. 10). Accordingly, the MS data provide clear evidence for the simultaneous modification of E310 of Src-K295M by amidation and ADP ribosylation. The exact molecular mechanism of the concurrent modifications remains to be uncovered. However, since Src-K295M/E310Q is no longer modified by ADP ribosylation (Fig. 4d), we postulate a one-step mechanism where amidation and ADP ribosylation are directly coupled to each other rather than a two-step mechanism with successive amidation and ADP ribosylation.

## Discussion

Here we show the modification and inactivation of the host cell kinase Src by a bacterial effector protein, resulting in the inhibition of a number of Src-mediated actin polymerization events including opsono-phagocytosis and pathogen-induced actin polymerization. EspJ targets the kinase domain of Src, disrupting Src autophosphorylation and the phosphorylation of Src substrates by post-translational modification of residue E310. Protein kinase domains are highly conserved sharing a common bilobal structure and 12 conserved motifs^24^. Phosphotransfer occurs in the cleft between the two lobes^25^, and E310 is part of the catalytic C helix, which projects into the catalytic cleft forming a salt bridge with K295, required for phosphotransfer^7^,^11^. Addition of ADP ribose on E310 would therefore disrupt salt-bridge formation and abrogate catalytic activity. By post-translationally modifying a highly conserved residue such as E310, EspJ may be able to inactivate multiple kinases (Supplementary Fig. 11). This is further supported by the fact that EspJ can inhibit EPEC and Vaccinia actin polymerization, which rely on both Src and Abl family kinases^19^,^20^.

Host cell tyrosine kinases are hijacked by many bacterial and viral pathogens during infection to enhance their own adhesion or spread, trigger internalization or ensure they remain extracellular. For example, *Shigella* initiates host cell signalling to induce membrane ruffles facilitating their invasion requiring Src and Abl/Arg kinases^26^,^27^. The *Helicobacter pylori* effector protein CagA is phosphorylated by SFK and Abl/Arg with pleiotropic effects within the cell, including cytoskeletal rearrangements and cell elongation^28^. Interestingly, phosphorylated CagA then initiates a negative-feedback loop to inhibit Src activity by activating Csk, which then phosphorylates Src on Y527 inducing the inactive conformation^29^. In this study, we show that in addition to using host cell kinases for actin pedestal formation, EPEC translocates an effector protein to inhibit Src signalling. Inhibition of Src, Abl and possibly further tyrosine kinases by EspJ could contribute to EPEC and EHEC virulence by blocking phagocytosis and pedestal formation by secondary EPEC infection, or by antagonizing Tir signalling and promoting pedestal disassembly during late stages of infection. However, given Src is required for many signalling events, the full role during infection requires further analysis.

The post-translational modification ADP ribosylation is used by many pathogens to target host cell signalling. For example, *Corynebacterium diptherium* diphtheria toxin inhibits protein synthesis by ADP-ribosylating elongation factor 2 (refs 30, 31). Several other bacterial toxins and effector proteins use ADP ribosylation to disrupt host cell cytoskeletal signalling. *P. aeruginosa* type-III secretion system effector ExoT ADP-ribosylates the Crk adaptor proteins^32^, which are involved in phagocytosis^33^, while the *Clostridium botulinum* ADP-ribosylating toxins C2 and C3 target monomeric actin and Rho GTPases, respectively^34^,^35^. The *P. syringae* effector HopF2, which shares homology with EspJ, ADP-ribosylates Mitogen-activated protein kinase (MAPK) disrupting plant responses to infection^36^. However, the activity of EspJ is novel as the combined amidation and ADP ribosylation of a target protein has not previously been reported. Furthermore, the inability to detect Src Q310 by MS and the lack of modification of Src E310Q suggest that amidation and ADP ribosylation are coupled rather than individual reactions. This represents a novel mechanism of action, the biochemical details of which require further investigation. EspJ therefore adds to the growing repertoire of bacterial effectors, which post-translationally modify host cell proteins, in this case using a novel amidase and ADP- ribosyltransferase activity to inactivate the tyrosine kinase Src.

## Methods

### Eukaryotic cell culture

Swiss 3T3, Cos-7 and J774.A1 cell lines were maintained in 4,500 mg l^−1^ glucose Dulbecco’s modified Eagle’s medium (DMEM) (Sigma) supplemented with 10% heat-inactivated fetal bovine serum and 2 mM Glutamax (Invitrogen) at 37 °C and 5% CO_2_.

### Bacterial strains and growth conditions

The bacterial strains used in this study are listed in Supplementary Table 1. Bacteria were cultured in Luria-Bertani (LB) broth at 37 °C, with ampicillin (100 μg ml^−1^), chloramphenicol (34 μg ml^−1^) or kanamycin (50 μg ml^−1^) as appropriate. For EPEC infections, overnight cultures were primed in DMEM by diluting 1:100 and incubating statically at 37 °C and 5% CO_2_ for 3 h as described^37^. For EHEC cultures, bacteria were grown in LB shaking for 8 h, then diluted 1:100 in DMEM and incubated overnight statically at 37 °C and 5% CO_2_. Bacterial cultures were induced with 0.05 mM isopropyl-beta-D-thiogalactopyranoside (IPTG) 30 min before infection if required.

EPEC JPN15 *espJ* mutant was constructed using the lambda red method^38^. Briefly, a PCR product was generated by amplifying the kanamycin resistance cassette from the pKD4 template plasmid using primers (EPEC-*espJ*-pKD4-f and EPEC-*espJ*-pKD4-r shown in Supplementary Table 2), which add 50 nucleotides of flanking DNA regions homologous to the 5′ and 3′ ends of the *espJ* gene. The PCR product was transformed into EPEC JPN15 containing the pKD46 plasmid. Clones were grown on LB medium containing kanamycin, the pKD46 cured by growth at 42 °C and the mutation verified by PCR using primers flanking *espJ* gene and primers into the antibiotic resistance gene.

### Plasmid construction

Oligonucleotides used for gene amplification and site-directed mutagenesis are shown in Supplementary Table 2, and plasmids used in this study are listed in Supplementary Table 3. For pcDNA–NTAP constructs, genes optimized for mammalian expression encoding EspJ_EHEC_, EspJ_EPEC_ and EspJ_EPEC_ R79A/D187A were synthesized by GeneArt and subcloned into pcDNA–NTAP. For all other constructs, bacterial sequences were amplified from EPEC O127:H6 E2348/69 genomic DNA. The QuikChange II Site-Directed Mutagenesis kit (Stratagene) was used as per the manufacturer’s instructions to generate pcDNA–NTAP-espJ_EHEC_ R79A/D187A and pSA10-espJ R79A, D187A and R79A/D187A. Mutated *espJ*_*EPEC*_was then subcloned into pET28a and pRK5. pCB6–Src expression constructs were a kind gift from Professor Michael Way. Full-length Src (chicken c-Src) and Src SH1 (250–533) sequences were subcloned into pEGFP-N1 for expression with a myc tag or pGEX–KG for expression as a GST fusion. Site-directed mutagenesis was used to insert E310A and E310Q mutations. All constructs were verified by DNA sequencing.

### Protein expression and purification

BL21 (pET28a-espJ_28-217_) strains were grown in LB overnight at 37 °C and then diluted 1:100 in LB or in minimal media containing 0.07% ^15^NH_4_Cl for ^1^H^15^N HSQC analysis. Cultures were grown until an OD_600_ of ~0.6, induced with 1 mM IPTG and grown for a further 18 h at 37 °C. For purification, the culture was centrifuged at 2,400 relative centrifugal force (RCF) for 20 min and the pellet resuspended in 25 ml denaturing protein buffer (8 M urea, 50 mM NaPO_4_ pH 7.4, 200 mM NaCl, 10 mM imidazole and 5 mM β-mercaptoethanol) with cOmplete EDTA-free protease inhibitors (Roche) and lysed with three passes through an Emulsiflex B-15. Samples were clarified at 17,000 RCF for 20 min and the supernatant loaded onto His-bind resin (Novagen) pre-equilibrated in denaturing protein buffer. Resin was washed with denaturing protein buffer, denaturing protein buffer containing 30 mM imidazole and the sample eluted with denaturing protein buffer containing 200 mM imidazole. Eluents were dialysed against 1 M urea, 50 mM NaPO_4_ pH 7.4, 200 mM NaCl and 5 mM β-mercaptoethanol followed by the same buffer containing no urea and finally gel filtered using a Superdex-75 gel filtration column (GE healthcare). His-tagged TirC_EPEC_ was purified from BL21(pET28a-TirC_EPEC_) grown in LB as above, except bacteria were grown at 30 °C for 4 h following IPTG induction and purification was performed on ice with non-denaturing buffers. TirC_EPEC_ was eluted in 5 ml protein buffer with 200 mM imidazole and dialysed against 50 mM Tris pH 7.5, 200 mM NaCl and 5 mM β-mercaptoethanol 10% glycerol. GST and GST-tagged Src derivatives were purified from BL21 carrying the appropriate pGEX–KG construct, following induction at 30 °C for 4 h using Glutathione Sepharose resin (GE Healthcare) as per manufacturers’ instructions.

### NMR analysis

NMR ^1^H^15^N HSQC experiments were performed on a Bruker Avance II 800 MHz spectrometer equipped with a TXI cryoprobe at 295 K using 0.25 mM ^15^N-labelled EspJ in 50 mM NaPO_4_ pH 7.4, 200 mM NaCl, 5 mM β-mercaptoethanol and 10% D_2_O in the presence and absence of NAD^+^ (Sigma) prepared in the same buffer. Data were processed with NMRpipe^39^ and analysed with NMRview^40^.

### Eukaryotic cell transfection

Cos-7 cells and Swiss 3T3 cells were seeded onto glass coverslips in 24-well plates at a density of 5 × 10^4^ or 7.5 × 10^4^ cells per well 24 h prior to transfection with FuGene 6 (Roche) or Lipofectamine 2000 (Invitrogen), respectively, according to the manufacturer’s instructions. Cells were incubated at 37 °C with 5% CO_2_ and assayed 15 h post transfection.

### EPEC/EHEC infection of eukaryotic cells

J774.A1 cells were seeded on glass coverslips in a 24-well plate at a density of 1.5 × 10^5^ cells per well and cultured overnight. Cells were starved in serum free (SF)-DMEM before infection with 200 μl JPN15 cultures, grown as described above. Plates were centrifuged at 500 RCF for 4 min and incubated for 1 h at 37 °C in 5% CO_2_. Infected cells were washed three times with PBS and challenged with opsonized RBCs to assay phagocytosis, as described below. Transfected Swiss 3T3 cells were infected with 100 μl EPEC or EPEC (pSA10-TccP) culture, grown as described above, and incubated for 1 or 3 h, respectively. For EHEC 85–170 infection, 25 μl culture was added and plates centrifuged at 500 RCF for 4 min, incubated at 37 °C in 5% CO_2_ for 2.5 h washed three times and incubated for a further 2.5 h. For all infections, cells were washed three times in PBS and fixed with 4% paraformaldehyde for 20 min at room temperature (RT).

### Vaccinia infection

Vaccinia expression vectors pEL-NTAP-espJ_EHEC_ and pEL-NTAP-espJ_EHEC_ R79A/D187A were generated by replacing GFP with the relevant insert in a previously described pEL vector^3^. For Vaccinia infection assays, HeLa cells were seeded on fibronectin-coated coverslips at ~50% confluency and cultured overnight. Cells were infected with a WR strain of Vaccinia virus expressing an RFP-tagged version of the viral core protein A3 (ref. 41). Approximately 4 h post infection, cells were transfected with indicated constructs or pEL-CFP (control) using the Fugene transfection protocol (Promega) and then fixed in 4% paraformaldehyde ~9 h post infection. Cells were permeabilized with PHEM buffer (60 mM PIPES, 25 mM Hepes, 10 mM EGTA and 1 mM Mg-acetate, pH 6.9), incubated with α-FLAG M2 (Sigma), washed with PBS, incubated with Alexa350 α-mouse and Alexa488 Phalloidin (Invitrogen) and mounted in mowiol.

### RBC opsonization and phagocytosis assay

0.3 or 0.1 μl of sheep RBC per well (J774.A1 or Cos-7, respectively) were opsonized with an equal volume of α-sRBC IgG (Sigma) previously diluted 1:50 in gelatin veronal buffer (Sigma) and rotated in a total volume of 500 μl gelatin veronal buffer for 30 min at RT. Opsonized RBCs were pelleted at 1,500 RCF for 2 min and resuspended in 500 μl DMEM per well. Prior to challenge, cells were serum-starved for at least 1 h. To assay for % internalization, infected cells were challenged with opsonized RBC for 30 min and transfected Cos-7 cells were challenged for 90 min. Where actin accumulation or phosphotyrosine staining were assessed, infected macrophages were incubated at 4 °C for 15 min and then 37 °C for 8 min after the addition of IgG-RBC. Where differential staining of RBC was required, cells were chilled on ice, washed with PBS and external RBC were stained with Alexa647 conjugated α-rabbit antibody (1:500, Invitrogen) before fixing with 4% paraformaldehyde for 20 min at RT.

### Immunofluorescence staining

Fixed cells were permeabilized with 0.1% Triton X-100 for 2 min and incubated in 2% bovine serum albumin (BSA)/PBS at RT to block non-specific interactions. Total RBC were then stained with Alexa555 conjugated α-rabbit antibody (1:500, Invitrogen). Alternatively, cells were incubated with antibodies against Flag M2 (1:500, Sigma), Myc tag clone 4A6 (1:500, Millipore), Tir (1:500), phosphotyrosine (1:500, Sigma), Nck (1:250, Millipore) or Src pY418 (labels chicken Src pY416) (1:500, Sigma) for 45 min at RT, washed and then incubated with appropriate secondary antibodies (1:200, Jackson Immunoresearch), Phalloidin TRITC (Sigma) or Phalloidin AlexaFluor350 (Invitrogen) to visualize F-actin and 4',6-diamidino-2-phenylindole (Invitrogen) to visualize cellular and bacterial nuclei. Coverslips were mounted in ProLong Gold antifade reagent (Invitrogen) and analysed using a ZEISS Axio Imager fluorescence microscope. The percentage of phagocytosis was defined as % bound RBC that are internalized by macrophages/transfected Cos-7 cells. In total, 50 transfected Cos-7 or 100 J774.A1 cells were counted for each experiment. For pedestal analysis, actin, Nck or pY416 staining was scored for bacteria attached to 50 transfected Swiss 3T3 cells. Three repeats were performed for each experiment. GraphPad Prism v6.0 (GraphPad Software, California, USA) was used to statistically analyse data sets using one-way analysis of variance, where appropriate. A significant result is defined as *P*<0.05 (shown as *, *P*<0.01 as **, *P*<0.001 as ***, and *P*<0.0001 as ****) as compared with uninfected, FcγRIIa or GFP control.

### Immunoprecipitations and kinase assay

For analysis of receptor phosphorylation 8 × 10^5^ Cos-7 cells were seeded 24 h before transfection with pEGFP-FcγRIIa or pEGFP-FcγRIIa Y282F/Y298F along with pcDNA–NTAP, pcDNA–NTAP-espJ_EPEC_ or pcDNA–NTAP-espJ_EPEC_ R79A/D187A. Cells were washed and incubated with serum-free media for 1 h before incubation with 0.5 μg ml^−1^ mouse anti-FcγRIIa (IV.3) (Stemcell Technologies) at 4 °C. Cells were washed three times and treated with 2 μg ml^−1^ goat anti-mouse antibody (Merck) for crosslinked or media alone for non-crosslinked samples. Samples were washed three times and lysed on ice in 1 ml RIPA buffer (1% Triton X-100, 0.5% Nonidet P-40, 100 mM NaCl, 2 mM EGTA, 2 mM EDTA and 30 mM HEPES, pH 7.4) with cOmplete EDTA-free protease inhibitors (Roche) and phosSTOP phosphatase inhibitors (Roche). Lysed cells were clarified by centrifugation at 510 RCF for 10 min and the supernatant was mixed with 20 μl protein G Dynabeads (Invitrogen) for 2.5 h at 4 °C and 30 min at RT. For non-crosslinked samples, beads were pre-coated with anti-mouse IgG. Beads were washed with RIPA buffer, 0.2% Triton X-100/tris buffered saline and tris-buffered saline, then boiled in 2 × Laemmli sample buffer for 5 min. Proteins were separated by SDS–PAGE and transferred to Hybond PVDF membrane (GE Healthcare) before blocking in 10 mM Tris pH 7.4, 150 mM NaCl, 0.1% Tween-20, 1 mM EDTA, 3% BSA and 0.5% gelatin. The membrane was probed with mouse anti-pTyr antibody (Sigma) diluted 1:2,500 and then HRP-conjugate anti-mouse (1:10,000, Jackson ImmunoResearch) and visualized with EZ-ECL (GeneFlow) as per the manufacturer’s instructions. Membranes were stripped with RestorePLUS stripping buffer (Thermo Scientific) and probed with rabbit anti-GFP (Abcam) diluted 1:1,000 in 3% milk, 0.1% Tween-20/PBS and then HRP-conjugated anti-rabbit antibody (1:10,000, Jackson ImmunoResearch).

For Src immunoprecipitation assays, 1.8 × 10^6^ Swiss 3T3 cells were seeded 24 h before transfection with pEGFP-N1-SrcY527F-myc or pEGFP-N1-Src 250–533 (SH1)-myc and pcDNA–NTAP, pcDNA–NTAP-espJ_EHEC_ or pcDNA–NTAP-espJ_EHEC_ R79A/D187A. After 16 h, cells were lysed in 250 μl lysis buffer (0.5% Triton X-100, 0.5% Nonidet P-40, 20 mM NaPO_4_ pH 7.4, 150 mM NaCl and 2 mM EGTA) with cOmplete EDTA-free protease inhibitors (Roche) and phosSTOP phosphatase inhibitors (Roche). Lysates were clarified at 10,000 RCF for 15 min and incubated with anti-Myc clone A46 (Millipore) antibody-coated protein G Dynabeads (Invitrogen) for 3 h at 4 °C. Bead complexes were washed three times with lysis buffer and twice in kinase assay buffer (100 mM Tris pH 7.2, 50 mM NaCl, 125 mM MgCl_2_, 25 mM MnCl_2_, 2 mM EGTA, 0.1 mg ml^−1^ BSA, 2 mM dithiothreitol and 250 μM Na_3_VO_4_) and the myc-tagged protein eluted in kinase buffer with 20 μg ml^−1^ Myc peptide (Sigma) for 30 min at 30 °C. Eluates were incubated with 2 μg TirC_EPEC_ and 100 μM adenosine triphosphate for 30 min at 30 °C, boiled in Laemmli sample buffer and analysed by SDS–PAGE and immunoblot as above.

### ADP-ribosylation assay

Purified His-tagged EspJ_28-217_ and EspJ_28-217_ R79A/D187A (0.5 μg) was incubated with 2 μg GST or GST-tagged Src, Src K295M, Src K295M/E310Q, K295M/E310A or SrcSH1 K295M in PBS pH 7.5 with 150 μM NAD^**+**^ and 0.5 μCi ^32^P-labelled NAD^**+**^. Samples were incubated at RT for 30 min, then separated by SDS–PAGE and analysed by phosphorimaging (Molecular Dynamics).

### Protein digest

For in-gel digestion, the excised gel bands were destained with 30% acetonitrile, shrunk with 100% acetonitrile and dried in a vacuum concentrator (Concentrator 5301, Eppendorf, Hamburg, Germany). Digests with trypsin, elastase and thermolysin were performed overnight at 37 °C in 0.05 M NH_4_HCO_3_ (pH 8). Approximately 0.1 μg of protease was used for one gel band. Peptides were extracted from the gel slices with 5% formic acid.

### NanoLC-MS/MS analysis

NanoLC-MS/MS analyses were performed on an LTQ-Orbitrap Velos Pro (Thermo Scientific) equipped with an EASY-Spray Ion Source and coupled to an EASY-nLC 1000 (Thermo Scientific). Peptides were loaded on a trapping column (2 cm × 75 μm inner diameter, PepMap C18 3 μm particles, 100 Å pore size) and separated on an EASY-Spray column (25 cm × 75 μm inner diameter, PepMap C18 2 μm particles, 100 Å pore size) with a 45-min linear gradient from 3 to 30% acetonitrile and 0.1% formic acid. MS scans were acquired in the Orbitrap analyzer with a resolution of 30,000 at *m*/*z* 400; MS/MS scans were acquired in the Orbitrap analyzer with a resolution of 7,500 at *m*/*z* 400 using HCD fragmentation with 30% normalized collision energy. A TOP5 data-dependent MS/MS method was used; dynamic exclusion was applied with a repeat count of 1 and an exclusion duration of 30 s; singly charged precursors were excluded from selection. Minimum signal threshold for precursor selection was set to 50,000. Predictive AGC was used with an AGC target value of 1e6 for MS scans and 5e4 for MS/MS scans. The same options were used for ETD fragmentation except for the following settings: a TOP3 method was applied, singly and doubly charged precursors were excluded, ETD activation time was set to 60 ms for triply and 45 ms for quadruply charged precursors and the AGC target was set to 300,000 for fluoranthene. Lock mass option was applied for internal calibration in all runs using background ions from protonated decamethylcyclopentasiloxane (*m*/*z* 371.10124).

Mascot Distiller 2.4 was used for raw data processing and for generating peak lists, essentially with standard settings for the Orbitrap Velos (high/high settings). Mascot Server 2.4 was used for database searching with the following parameters: peptide mass tolerance: 8 p.p.m., MS/MS mass tolerance: 0.02 Da, enzyme: ‘trypsin’ with three missed cleavage sites allowed for trypsin or ‘none’ for elastase and thermolysin; fixed modification: carbamidomethyl (C), variable modifications: Gln->pyroGlu (N-term. Q), oxidation (M) and ADP ribosylation (RKCEDNQ). Database searching was performed against a small custom database containing Src sequence (K295M and K295M+E310Q).

## Author contributions

J.C.Y., A.C., A.E.L., J.A.G., J.P., A.S. and D.J.B. designed and performed the experiments. J.C.Y. performed phagocytosis, transfection and infection experiments. D.J.B. performed Vaccinia virus experiments and J.A.G., A.E.L. and A.S. performed the *in vitro* ADP-ribosylation experiments. J.C.Y., A.C., M.W. and G.F. wrote the manuscript. D.M., A.A., A.M., A.S., A.E.L., S.J.M., M.W., K.A., E.L.H. and G.F. contributed to experimental design and manuscript preparation.

## Additional information

**How to cite this article:** Young, J. C. *et al*. The *Escherichia coli* effector EspJ blocks Src kinase activity via amidation and ADP ribosylation. *Nat. Commun.* 5:5887 doi: 10.1038/ncomms6887 (2014).

**Accession codes:** The mass spectrometry proteomics data have been deposited to the ProteomeXchange Consortium via the PRIDE partner repository with the data set identifier PXD001461 and 10.6019/PXD001461.

## Supplementary Material

Supplementary Information Supplementary Figures 1-11, Supplementary Tables 1-4, and Supplementary References
