# Supplementary material for: The Escherichia coli effector EspJ blocks Src kinase activity via amidation and ADP ribosylation
Source: Nat Commun. 2014 Dec 19;5:5887. doi: 10.1038/ncomms6887 (PMC4284639; doi:10.1038/ncomms6887)
Supplement: Supplementary Information — Supplementary Figures 1-11, Supplementary Tables 1-4, and Supplementary References [file ncomms6887-s1.pdf]

Supplementary Materials

Supplementary Figures 1-11

Supplementary Tables 1-4

Supplementary References

Supplementary Figures

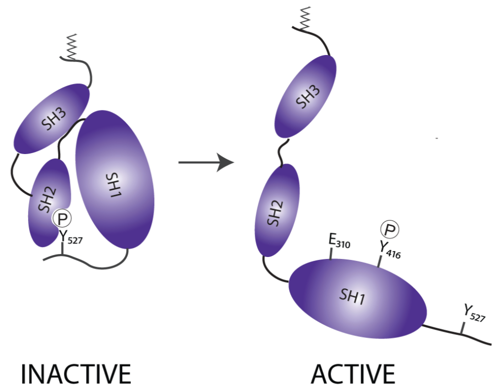

Supplementary Figure 1. **Schematic illustration of the inactive and active conformations of SFKs.** In the inactive state the SH2 and SH3 domains are packed against the catalytic SH1 domain. Phosphorylation of the C-terminal Y527 facilitates this conformation through interaction with the SH2 domain. Dephosphorylation of Y527 and/or competitive interactions of ligands with the SH2 and SH3 domains disrupts this conformation, opening the kinase. Autophosphorylation at Y416 promotes the open conformation and kinase activity. The relative position of E310 is shown.

```
EspJ_EPEC         MPIIKNCLSSISNILRNEKTSYSLIKTEQTGKLLNRKITTTPTPAKLLSYRNADLIKENY 60
EspJ_EHEC         MSIIKNCLSLINNALNIQKTSYSLTKMEQAGKLLNRKITPENTPPMLLSYRNADLTQEKN 60
AvrPphF           -------MGNICNSGGVSRT-YSPPTSPVYGSGVSSPSRFVGQYTLISIHQLSSEERENF 52
                         :. * *    .:* **  .    *. :.         . :  :: :.  :*:

EspJ_EPEC         ITEK-VLSIFNIKRDFVAVRIQSNQFTDLKNKTIQGHKDTVAKVIDWYNPQKNAFGIMMG 119
EspJ_EHEC         ITER-VLSIFKIKRDFVAVRIQNNQFTDLKNKKIQGHQNTVASVMDWYNPQKNALGITMG 119
AvrPphF           LDAHDPMRVYDLNSETSVYRTTQREY--VRNGYATGNPNSGAIIALHEELQESPYAQHIG 110
                  :  :  : ::.:: :  . *  ..::  ::*    *: :: * :    : *:.. .  :*

EspJ_EPEC         TPRRSADIAKEESRNALNFMIMEKDTFNEKILNSNANLQKKYGTTENSSWVSASVGSLLD 179
EspJ_EHEC         TPRKSADIAKEEHRNALNFMIMEKNTFHEKILNSNDNLQKSYSKTEDSSWVAASVGSLLD 179
AvrPphF           ARPDQADAYRPRTAHAS---SLNTPSLNVMAGQGALSALRGYAGS-DHVTTEMRLGDFLD 166
                  :   .**  : .  :*     ::. :::    :.  .  : *. : :   .   :*.:**

EspJ_EPEC         KGAKVYPDISCSLRLG---EPFIITLPETVRLDVNIHPLKK 217
EspJ_EHEC         KGAKVYPDTSCSLRLG---EPFIFTLPESVRVDVDIYPLKK 217
AvrPphF           QGGKVYSDTSAMSAGGDSVEALIVTLPKGRKVPVNILD--- 204
                  :*.***.* *.    *   *.:*.***:  :: *:*
```

Supplementary Figure 2. Sequence alignment of EspJ with AvrPphF. EspJ amino acid sequence from EPEC O127:H6 E2348/69 and EHEC O157:H7 (Sakai) aligned with AvrPphF_ORF2 from *Pseudomonas syringae* pv. *phaseolicola.* R79 and D187 are shown in bold and black. Amino acid color code: Red: small, hydrophobic, aromatic, not Y; Blue: acidic; Magenta: basic; Green: hydroxyl, amine, amide, basic; "*": identical; ":": conserved substitutions (same colour group); ".": semi-conserved substitution (similar shapes).

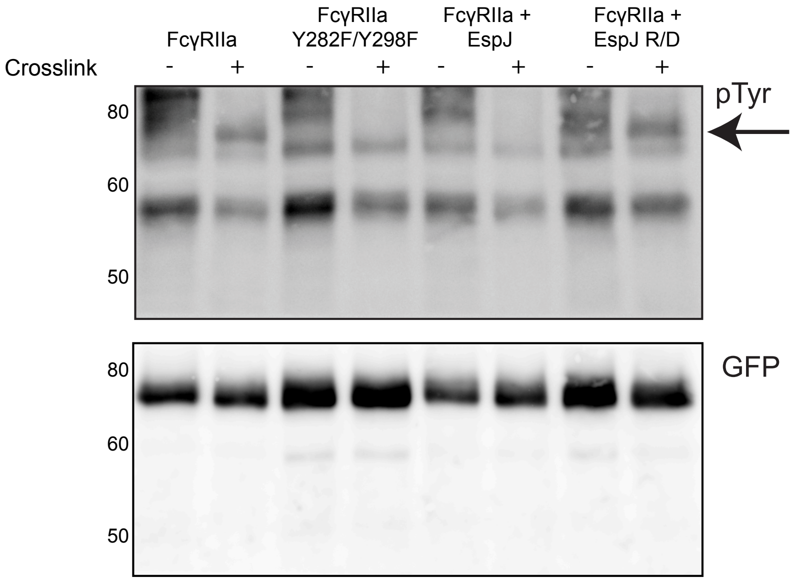

Supplementary Figure 3. EspJ inhibits FcγRIIa tyrosine phosphorylation. Full immunoblots corresponding to cropped versions shown in Fig. 2e. Cos-7 cells co-expressing GFP-tagged FcγRIIa or FcγRIIa Y282F/Y298F with EspJ, EspJ-R/D or an empty vector were treated with anti-FcγR IV.3 antibody with or without secondary antibody crosslinking. The FcγRIIa was immunoprecipitated and analyzed by immunoblotting with anti-pTyr and anti-GFP antibodies, which shows phosphorylation of wild type FcγRIIa in the control and EspJ-R/D-expressing cells (arrow) but not in cells expressing EspJ or FcγRIIa Y282F/Y298F. Similar results were obtained in three independent experiments. Representative immunoblots are shown.

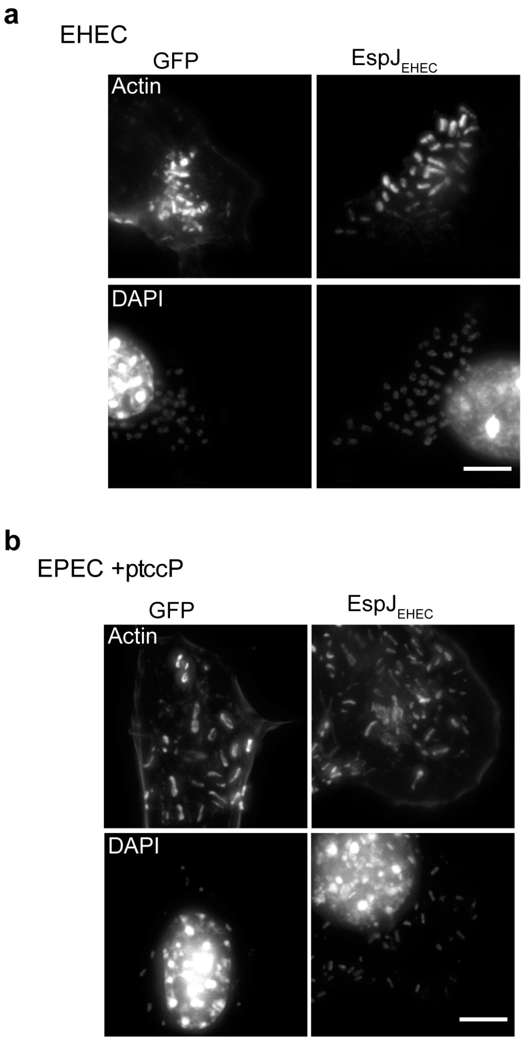

Supplementary Figure 4. EspJ does not affect EspF$_U$/TccP dependent pedestal formation. Swiss 3T3 cells expressing Flag-tagged GFP or EspJ$_{EHEC}$ were infected EHEC 85-170 **(a)** or EPEC E2348/69 expressing TccP from a plasmid **(b)**. Cells were stained with Phalloidin for actin visualization and DAPI to detect bacteria. Representative images are shown. Similar results observed in two independent experiments. Scale bar indicates 10μm.

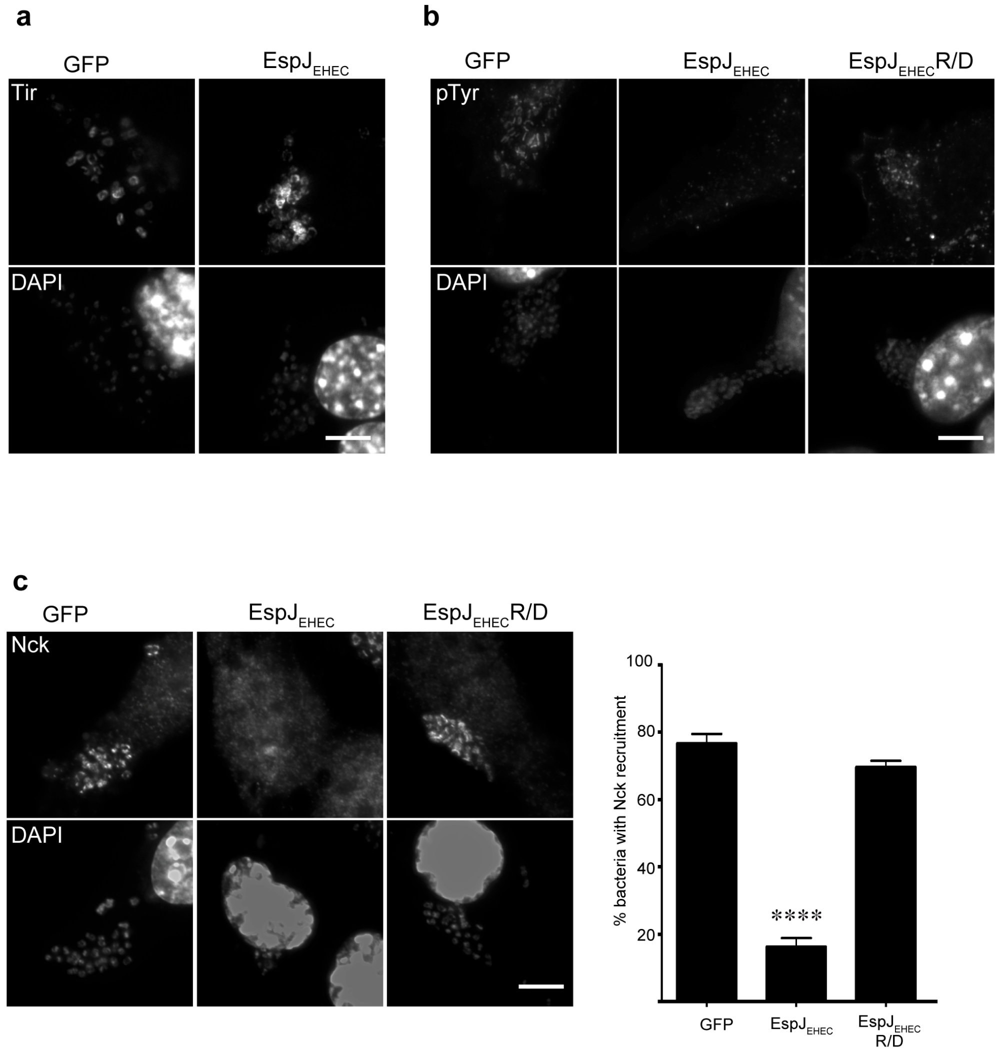

Supplementary Figure 5. EspJ inhibits tyrosine phosphorylation and Nck recruitment to the EPEC attachment sites dependent on its ART domain. Swiss 3T3 cells expressing Flag-tagged GFP, EspJ or EspJ-R/D were infected with EPEC E2348/69. Cells were stained with anti-Tir (a), anti-pTyr (b) or anti-Nck (c) and DAPI to visualize bacteria. Representative images are shown. Nck recruitment was quantified for bacteria attached to 50 transfected cells. Results are the mean ± SD of three independent experiments. Data sets were analysed using one-way ANOVA (GraphPad Prism v6.0) . A significant result is defined as $p < 0.05$ (shown as *, $p < 0.01$ as **, $p < 0.001$ as *** and $p < 0.0001$ as ****) as compared to GFP expressing cells. Scale bars indicate 10 µm.

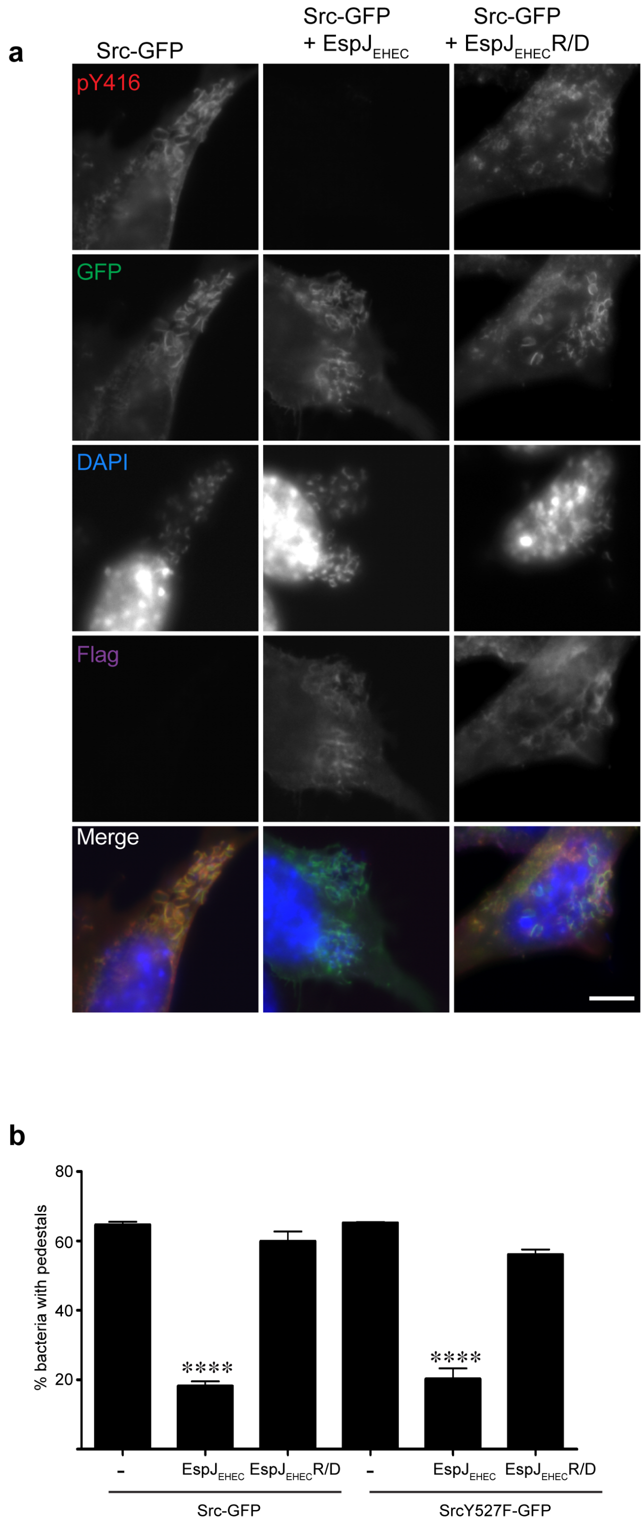

Supplementary Figure 6. EspJ inhibits recruitment of active Src to sites of bacterial attachment. (a) Swiss 3T3 cells were co-transfected with Src-GFP with an empty vector, Flag-tagged EspJ or EspJ-R/D and infected with EPEC. Immunofluorescent analysis with anti-pY416 (red), anti-Flag (purple) and DAPI (blue) revealed that although Src-GFP is recruited beneath EPEC attachment sites in all conditions, it is not activated in cells specifically expressing WT

EspJ. (b) Cells co-expressing Flag-tagged EspJ or EspJ-R/D, with Src-GFP or Src Y527F-GFP were infected with EPEC. Actin polymerization was quantified for bacteria attached to 50 transfected cells and was reduced by EspJ in cells expressing either wild type Src or constitutively active Src. Results are the mean ± SD of three independent experiments in which 50 cells were analyzed. Data sets were analysed using one-way ANOVA (GraphPad Prism v6.0) . A significant result is defined as $p< 0.05$ (shown as *, $p< 0.01$ as **, $p< 0.001$ as *** and $p< 0.0001$ as ****) as compared to GFP expressing cells. Scale bars indicate 10 μm.

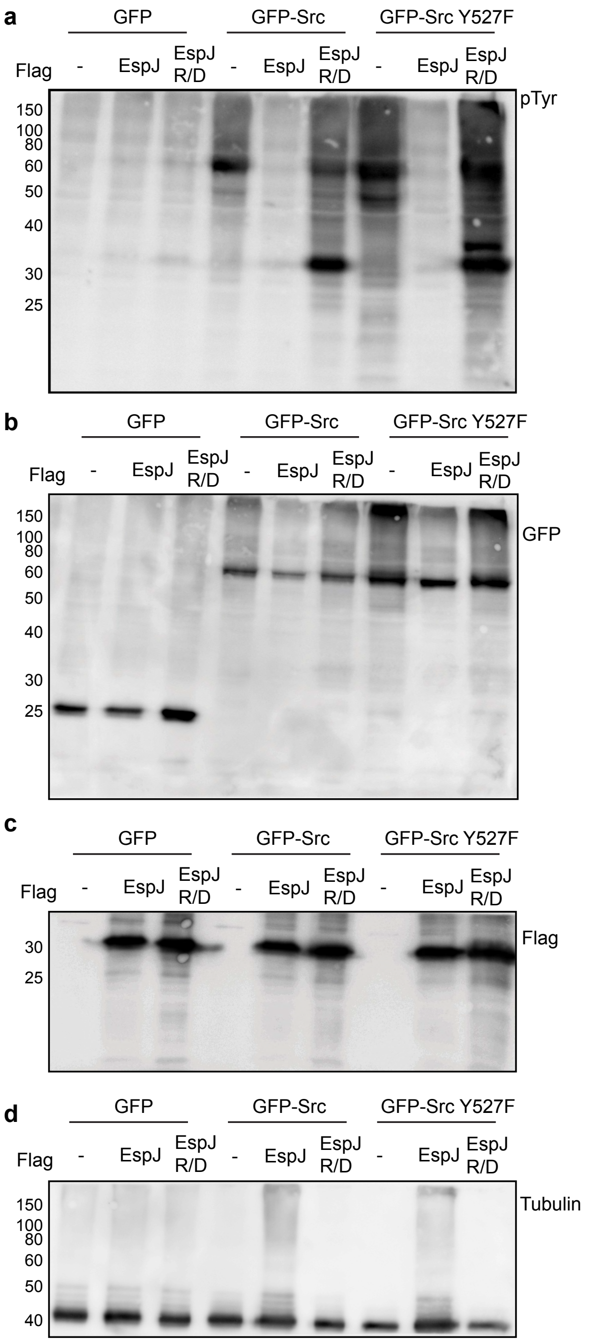

Supplementary Figure 7. EspJ inhibits Src dependent phosphorylation. Full immunoblots corresponding to cropped versions shown in Fig. 3d. Swiss 3T3 cells transfected with an empty vector or expressing Flag-tagged EspJ, or EspJ-R/D , were co-transfected with GFP, Src-GFP or Src Y527F-GFP and analyzed by anti-pTyr (a), anti-GFP (b), and anti-Flag (c) immunoblot. Anit-tubulin immunoblot shown as loading control (d). EspJ inhibited general protein tyrosine phosphorylation. Similar results were obtained in three independent experiments. Representative immunoblots are shown.

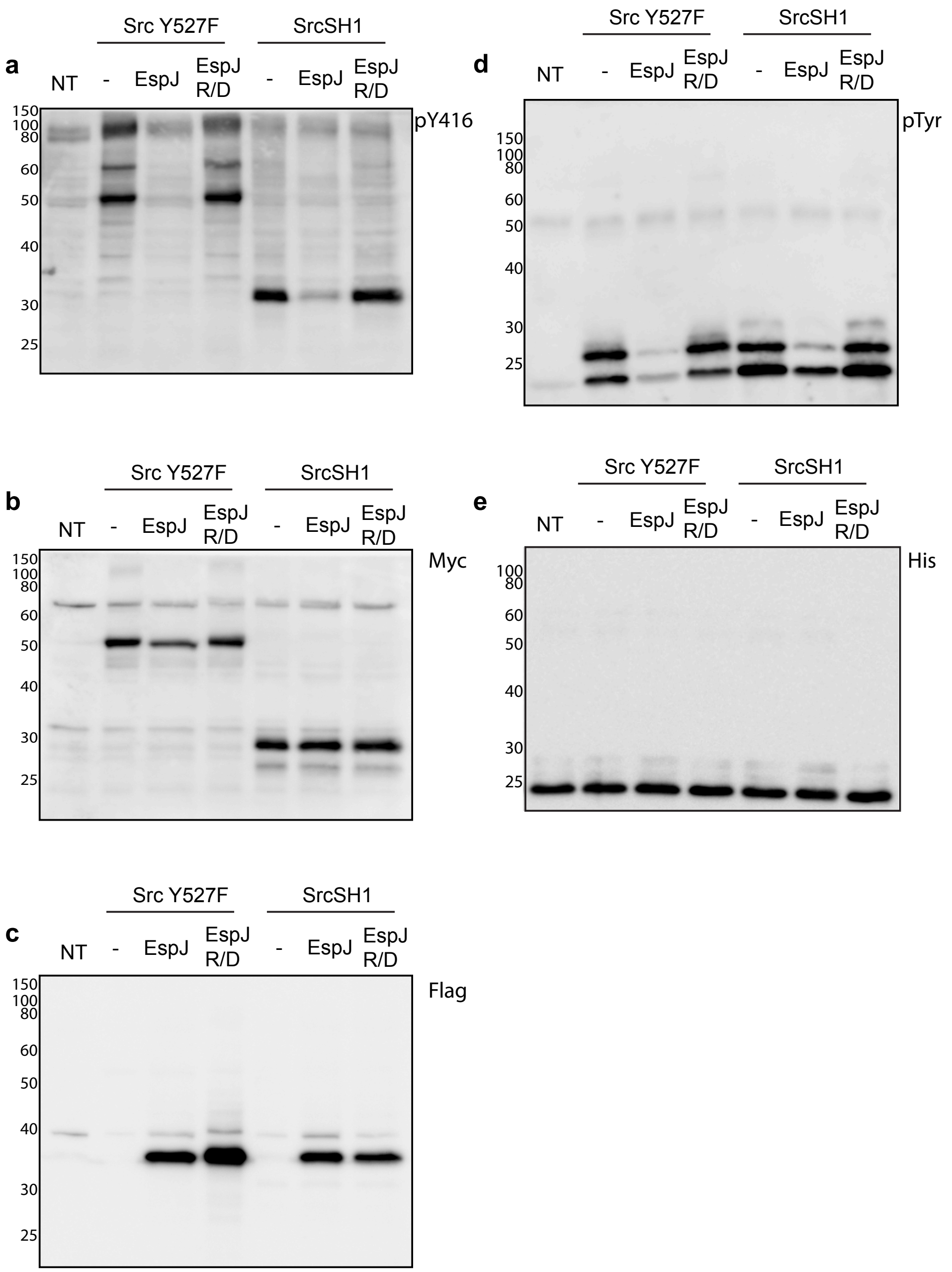

Supplementary Figure 8. EspJ inhibits Src kinase activity. Full immunoblots corresponding to cropped versions shown in Fig. 4a & b. Swiss 3T3 cells expressing myc-tagged Src Y527F or Src SH1 with Flag-tagged EspJ, or EspJ-R/D were analyzed by anti-pY416 (a), anti-Myc (b) and anti-Flag (c) antibodies. EspJ inhibited auto-phosphorylation (pY416) of both Src Y527F and Src SH1. Myc-tagged Src Y527F or Src SH1 were immunoprecipitated from lysates of cells expressing EspJ or EspJ and incubated with His-tagged $TirC_{EPEC}$. Analysis by immunobloting with anti-pTyr (d) and anti-His (e) antibodies indicated that Src Y527F and Src SH1 were inactive when immunoprecipitated from cells co-expressing EspJ. Similar results were obtained in three independent experiments.

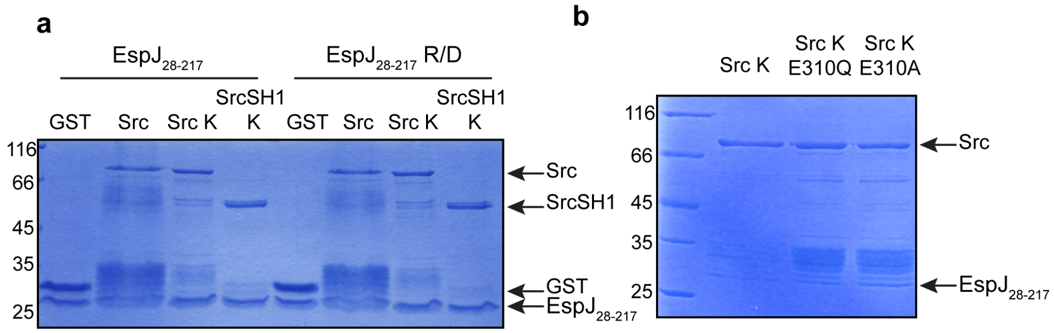

Supplementary Figure 9. EspJ ADP-ribosylates Src and SrcSH1 but not Src E310A or E310Q. Coomassie stained gels corresponding to the autoradiographs shown in Fig. 4c &d. (a) Recombinant EspJ and EspJ-R/D were incubated with GST, GST-Src, GST-Src K295M (Src K) or GST-SrcSH1 K295M (SrcSH1 K) and ^{32}P-labeled NAD^{+}. (b) Recombinant EspJ was incubated with Src K295M, Src K295M/E310Q or Src K295M/E310A and ^{32}P-labeled NAD^{+}. Arrows indicating positions of the purified proteins.

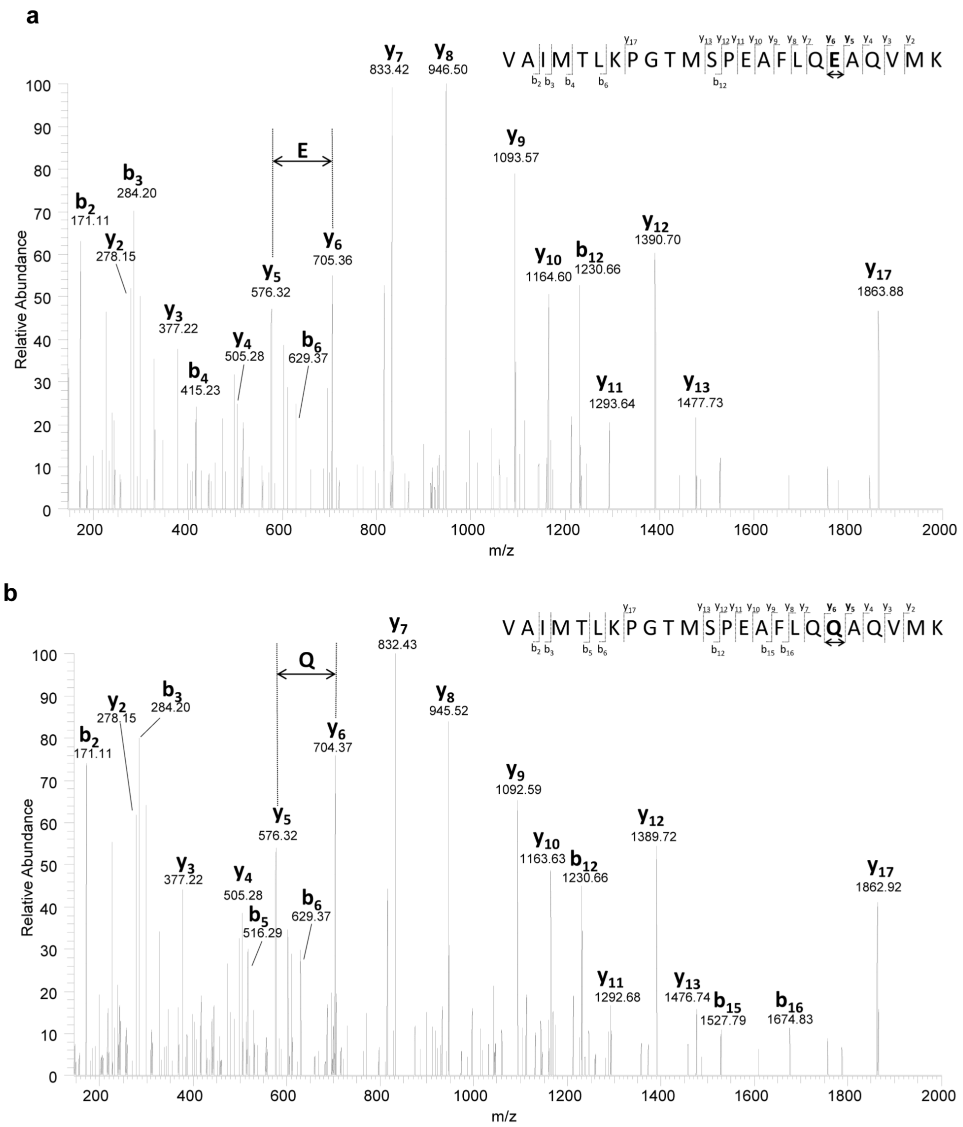

Supplementary Figure 10. EspJ converts Src Glu310 to Gln (a) HCD fragment ion spectrum of the precursor 874.1254 (3+). Database searching identified the tryptic peptide with the sequence VAIMTLKPGTMSPEAFLQEAQVMK. The spectrum indicated a Glu residue was present at position 310 in SrcK295M. (b) HCD fragment ion spectrum of the precursor 873.7925 (3+). Database searching identified the tryptic peptide with the sequence VAIMTLKPGTMSPEAFLQQAQVMK. The spectrum indicated a Gln residue was present at position 310 in SrcK295M treated with EspJ.

```
Abl       ITMKHKLGGGQYGEVYEGVWKKY---SLTVAVKTLKEDTME---VEEFLKEAAVMKEIKH 54
Arg       ITMKHKLGGGQYGEVYVGVWKKY---SLTVAVKTLKEDTME---VEEFLKEAAVMKEIKH 54
Src       LRLEVKLGQGCFGEVWMGTWNG----TTRVAIKTLKPGTMS---PEAFLQEAQVMKKLRH 53
Yes       LRLEVKLGQGCFGEVWMGTWNG----TTKVAIKTLKPGTMM---PEAFLQEAQIMKKLRH 53
Fyn       LQLIKRLGNGQFGEVWMGTWNG----NTKVAIKTLKPGTMS---PESFLEEAQIMKKLKH 53
Lyn       IKLVKRLGAGQFGEVWMGYYNN----STKVAVKTLKPGTMS---VQAFLEEANLMKTLQH 53
FAK       IELGRCIGEGQFGDVHQGIYMSPENPALAVAIKTCKNCTSDS-VREKFLQEALTMRQFDH 59
Syk       TLEDKELGSGNFGTVKKGYYQMKK-VVKTVAVKILKNEANDPALKDELLAEANVMQQLDN 59
                :* * :* *  * :         **:*  *  :      : :* **  *: : :

Abl       PNLVQLLGVCTREPPFYIITEFMTYGNLLDYLRECNRQEVNAVVLLYMATQISSAMEYLE 114
Arg       PNLVQLLGVCTLEPPFYIVTEYMPYGNLLDYLRECNREEVTAVVLLYMATQISSAMEYLE 114
Src       EKLVQLYAVVS-EEPIYIVTEYMSKGSLLDFLKGETGKYLRLPQLVDMAAQIASGMAYVE 112
Yes       DKLVPLYAVVS-EEPIYIVTEFMSKGSLLDFLKEGDGKYLKLPQLVDMAAQIADGMAYIE 112
Fyn       DKLVQLYAVVS-EEPIYIVTEYMNKGSLLDFLKDGEGRALKLPNLVDMAAQVAAGMAYIE 112
Lyn       DKLVRLYAVVTREEPIYIITEYMAKGSLLDFLKSDEGGKVLLPKLIDFSAQIAEGMAYIE 113
FAK       PHIVKLIGVIT-ENPVWIIMELCTLGELRSFLQVRKYS-LDLASLILYAYQLSTALAYLE 117
Syk       PYIVRMIGICE-AESWMLVMEMAELGPLNKYLQ--QNRHVKDKNIIELVHQVSMGMKYLE 116
            :* : .:     .  :: *    * * .:*:      :    ::    *:: .: *:*

Abl       KKNFIHRDLAARNCLVGENHLVKVADFGLSRLMTGDT--YTAHAGAKFPIKWTAPESLAY 172
Arg       KKNFIHRDLAARNCLVGENHVVKVADFGLSRLMTGDT--YTAHAGAKFPIKWTAPESLAY 172
Src       RMNYVHRDLRAANILVGENLVCKVADFGLARLIEDNE--YTARQGAKFPIKWTAPEAALY 170
Yes       RMNYIHRDLRAANILVGENLVCKIADFGLARLIEDNE--YTARQGAKFPIKWTAPEAALY 170
Fyn       RMNYIHRDLRSANILVGNGLICKIADFGLARLIEDNE--YTARQGAKFPIKWTAPEAALY 170
Lyn       RKNYIHRDLRAANVLVSESLMCKIADFGLARVIEDNE--YTAREGAKFPIKWTAPEAINF 171
FAK       SKRFVHRDIAARNVLVSSNDCVKLGDFGLSRYMEDST--YYKASKGKLPIKWMAPESINF 175
Syk       ESNFVHRDLAARNVLLVTQHYAKISDFGLSKALRADENYYKAQTHGKWPVKWYAPECINY 176
            .::***: : * *:      *:.****:: :  .   *     .* *:** ***.  :

Abl       NKFSIKSDVWAFGVLLWEIATYGMSPYPGIDLSQVYELLEKDYRMERPEGCPEKVYELMR 232
Arg       NTFSIKSDVWAFGVLLWEIATYGMSPYPGIDLSQVYDLLEKGYRMEQPEGCPPKVYELMR 232
Src       GRFTIKSDVWSFGILLTELTTKGRVPYPGMVNREVLDQVERGYRMPCPPECPESLHDLMC 230
Yes       GRFTIKSDVWSFGILQTELVTKGRVPYPGMVNREVLEQVERGYRMPCPQGCPESLHELMN 230
Fyn       GRFTIKSDVWSFGILLTELVTKGRVPYPGMNNREVLEQVERGYRMPCPQDCPISLHELMI 230
Lyn       GCFTIKSDVWSFGILLYEIVTYGKIPYPGRTNADVMTALSQGYRMPRVENCPDELYDIMK 231
FAK       RRFTSASDVWMFGVCMWEILMHGVKPFQGVKNNDVIGRIENGERLPMPPNCPPTLYSLMT 235
Syk       YKFSSKSDVWSFGVLMWEAFSYGQKPYRGMKGSEVTAMLEKGERMGCPAGCPREMYDLMN 236
            *:  **** **:   *    *  *: *    :*   :... *:     **  ::.:*

Abl       ACWQWNPSDRPSFAEIHQAF----- 252
Arg       ACWKWSPADRPSFAETHQAF----- 252
Src       QCWRKEPEERPTFEYLQAFLEDYF- 254
Yes       LCWKKDPDERPTFEYIQSFLEDYF- 254
Fyn       HCWKKDPEERPTFEYLQSFLEDYF- 254
Lyn       MCWKEKAEERPTFDYLQSVLDDFY- 255
FAK       KCWAYDPSRRPRFTELKAQLSTIL- 259
Syk       LCWTYDVENRPGFAAVELRLRNYYY 261
           **  .   ** *   .  :
```

Supplementary Figure 11. Alignment of the catalytic domain of tyrosine kinases. SH1 domain of Src (P12931,270-523), Fyn (P0624, 271-524), Yes (P07947, 277-530), Lyn (P07948, 247-501), Abl1 (P00519, 242-493), Arg (P42684, 288-539), Syk (P43405,371-631), and Fak1 (Q05397, 422-680) tyrosine kinases from *Homo sapiens*. Residues equivalent to Src (chicken) E310 are shown in bold and black showing the residue targeted by EspJ is conserved. Amino acid color code: Red: small, hydrophobic, aromatic, not Y; Blue: acidic; Magenta: basic; Green: hydroxyl, amine, amide, basic; "*": identical; ":": conserved substitutions (same colour group); ".": semi-conserved substitution (similar shapes).

Supplementary Tables 1-4

Supplementary Table 1. List of bacterial strains.

ICC No.	Name	Description	Source/Ref
	BL21 (DE3)	*E. coli* F– *omp*T *hsd*SB(rB–, mB–) *gal dcm* (DE3)	Invitrogen
ICC481	E2348/69	EPEC O127:H6	1
ICC715	E2348/69 pTccP	E2348/69 carrying pSA10-tccP	2
	JPN15	Plasmid (EAF) cured derivative O127:H6 E2348/69	3
ICC263	JPN15 Δ*espJ*	JPN15 *espJ* deletion mutant	This study
ICC608	85-170	EHEC O157:H7 spontaneous *stx*1- *stx2*-, NalR	4

Supplementary Table 2. List of oligonucleotides. Restriction sites shown in bold and mutated bases underlined.

Name	oligonucleotide sequence 5'-3'
pKD4_ espJ$_{EPEC}$ F	ccaatcataaagaactgcttatcatcaattagtaacatattacgcaacgagt gtaggctggagctgcttc
pKD4_espJ$_{EPEC}$ R	catccagcctgactgtttctggaagtgtaataatgaatggttctcccagtcat atgaatatcctccttag
pET28a_*Nde*I espJ28-217$_{EPEC}$ F	gatcatc**catatg**gagcaaacaggaaaactgttaaac
pET28a_espJ$_{EPEC}$ *Hind*III R	aat**aagctt**tcatttttgagtgggtggatattaac
pGEX-KG_*BamH*I src F	gt**ggatcc**atggggagcagcaagagc
pGEX-KG_*BamH*I src250 F	gt**ggatcc**ccacagacccagggactcgc
pGEX-KG_src *EcoR*I R	tc**gaattc**ttataggttctctccaggctgg
pSA10_*EcoR*I espJ$_{EPEC}$ F	ca**gaattc**atgccaatcataaagaactgcttat
pSA10_espJ$_{EPEC}$ *Pst*I R	ttgtcgaagcttgg**ctgcag**ctaagcgtagtctgggacgtcatatgggtat gctcctttttgagtgggtggatattaac
pRK5_espJ$_{EPEC}$ *BamH*I F	ctg**ggatcc**atgccaatcataaagaactgcttatc
pRK5_espJ$_{EPEC}$ *Pst*I R	tggcggccaagctt**ctgcag**tcatttttgagtgggtggatattaac
SDM_espJ$_{EPEC}$ R79A F	cgggatttcgttgctgtagcaatccaaagtaatcag
SDM_espJ$_{EPEC}$ D187A F	ggagccaaagtatatccagctatatcatgctctctgaga
SDM_espJ$_{EHEC}$ opt R79A F	ggacttcgtggccgtggccatccagaacaatcagttcacc
SDM_espJ$_{EHEC}$ opt D187A F	gccaaggtgtaccccgccaccagctgctccctg
pEGFP-N1_*EcoRI* src F	gt**gaattc**atggggagcagcaagagc
pEGFP-N1_*EcoRI* src250 F	gt**gaattc**atgccacagacccagggactcg
pEGFP-N1_src-myc-*BamH*I R	atggatccctacaggtcctcctcggagatcagcttctgctccgagccgga gcctaggttctctccaggc
SDM_src E310A F	ggaggccttcctgcaggcagcccaagtgatgaag
SDM_src E310Q F	ggaggccttcctgcagcaagcccaagtgatgaag

Supplementary Table 3. List of plasmids.

pICC No.	Name	Description	Source
pICC853	pET28a	Bacterial expression vector with N-terminal 6xHis tag	Invitrogen
pICC1611	pET28a-espJ	pET28a derivative encoding $EspJ_{EPEC}$	This study
pICC1612	pET28a-espJ R79A	pET28a derivative encoding $EspJ_{EPEC}$ R79A	This study
pICC1613	pET28a-espJ D187A	pET28a derivative encoding $EspJ_{EPEC}$ D187A	This study
pICC1614	pET28a-espJ R79A/D187A	pET28a derivative encoding $EspJ_{EPEC}$ R79A/D187A	This study
pICC609	pET28a-$tirC_{EPEC}$	pET28a derivative encoding carboxy terminus (residues 369–550) of Tir_{EPEC}	5
	pGEX-KG	Bacterial expression vector as GST fusion protein	6
pICC1615	pGEX-KG-src	pGEX-KG derivative encoding Src (chicken)	This study
pICC1616	pGEX-KG-src K295M	pGEX-KG derivative encoding kinase dead Src (chicken)	This study
pICC1617	pGEX-KG-srcSH1 K295M	pGEX-KG derivative encoding kinase dead Src 250-533	This study
pICC1633	pGEX-KG-src K295M/E310Q	pGEX-KG derivative encoding kinase dead Src and E310Q	This study
pICC1634	pGEX-KG-src K295M/E310A	pGEX-KG derivative encoding kinase dead Src and E310A	This study
pICC1223	pACYC-GFP	pACYC184 derivative encoding GFP	Olivier Marches
	pSA10	pKK17.1 containing *lacI gene*	1
pICC1618	pSA10-espJ	pSA10 derivative encoding $EspJ_{EPEC}$	This study
pICC1619	pSA10-espJ C7A	pSA10 derivative encoding $EspJ_{EPEC}$ C7A	This study
pICC1620	pSA10-espJ R79A	pSA10 derivative encoding $EspJ_{EPEC}$ R79A	This study
pICC1621	pSA10-espJ D187A	pSA10 derivative encoding $EspJ_{EPEC}$ D187A	This study
pICC1622	pSA10-espJ R79A/D187A	pSA10 derivative encoding $EspJ_{EPEC}$ R79A D187A	This study
pICC281	pSA10-tccP	pSA10 derivative encoding TccP	2

	pEGFP-FcγRIIa	GFP-tagged FcRIIa for expression in mammalian cells	7
	pEGFP-FcγRIIa Y282F/Y298F	pEGFP-FcγRIIa derivative with Y282F/Y298F mutations	7
	pRK5	N-terminal myc-tag mammalian expression vector	Clontech
pICC1623	pRK5-espJ	pRK5 derivative encoding myc-tagged $EspJ_{EPEC}$ for expression in mammalian cells	This study
pICC1624	pRK5-espJ R79A/D187A	pRK5 derivative encoding myc-tagged $EspJ_{EPEC}$ R79A D187A for expression in mammalian cells	This study
pICC1625	pcDNA-NTAP	pcDNA3.1 derivative for expression in mammalian cells with TAP-tag (3xFLAG-tag - TEV cleavage site – calmodulin binding peptide).	This study
pICC569	pcDNA-NTAP-GFP	pcDNA-NTAP derivative encoding TAP-tagged eGFP in pcDNA3.1(+)	8
pICC1626	pcDNA-NTAP-$espJ_{EPEC}$	pcDNA-NTAP derivative encoding codon optimized $EspJ_{EPEC}$	This study
pICC1627	pcDNA-NTAP-$espJ_{EPEC}$ R79A/D187A	pcDNA-NTAP derivative encoding codon optimized $EspJ_{EPEC}$ R79A D187A	This study
pICC1628	pcDNA-NTAP-$espJ_{EHEC}$	pcDNA-NTAP derivative encoding codon optimized $EspJ_{EHEC}$	This study
pICC1629	pcDNA-NTAP-$espJ_{EHEC}$ R79A/D187A	pcDNA-NTAP derivative encoding codon optimized $EspJ_{EHEC}$ R79A D187A	This study
	pCB6-Src-GFP	GFP-tagged Src for expression in mammalian cells	9
	pCB6-Src Y527F-GFP	GFP-tagged constitutively active Src Y527F for expression in mammalian cells	9
pICC1630	pEGFP-Src-myc	pEGFP-N1 derivative encoding myc-tagged Src	This study
pICC1631	pEGFP-Src Y527F-myc	pEGFP-N1 derivative encoding myc-tagged Src Y527F	This study
pICC1632	pEGFP-Src SH1-myc	pEGFP-N1 derivative encoding myc-tagged Src 250-533	This study

Supplementary Table 4: ADP-ribosylated peptides identified by database searching with Mascot. All peptides have been mapped exclusively to the sequence of Src-K295M/E310Q.

sequence	# methionine oxidations	Mascot PTM site probability [%]	m/z exp	Mr exp (Da)	Δm (ppm)	Mascot Score	fragmentation mode	protease	position
VAIMTLKPGTMSPEAFLQ**Q**AQVMK	0	51	1054.1484	3159.4234	-0.36	31	HCD	trypsin	292-315
VAIMTLKPGTMSPEAFLQ**Q**AQVMKK	0	55	1096.8455	3287.5147	-1.46	45	HCD	trypsin	292-316
VAI**M**TLKPGTMSPEAFLQ**Q**AQVMKK	1	21	1102.1776	3303.511	-1.03	19	HCD	trypsin	292-316
VAIMTLKPGTMSPEAFLQ**Q**AQVMK	0	74	790.8618	3159.4182	-1.99	63	ETD	trypsin	292-315
VAI**M**TLKPGTMSPEAFLQ**Q**AQVMK	1	20	794.8588	3175.4062	-4.18	15	ETD	trypsin	292-315
VAIMTLKPGTMSPEAFLQ**Q**AQVMKK	0	36	822.8862	3287.5158	-1.11	28	ETD	trypsin	292-316
KPGTMSPEAFLQ**Q**A	0	69	1023.4071	2044.7996	-0.3	20	HCD	elastase	298-311
KPGTMSPEAFLQ**Q**AQVM	0	44	1202.4902	2402.9658	-0.79	33	HCD	elastase	298-314
LQ**Q**AQVMKK	0	50	538.8948	1613.6626	-2.99	36	ETD	thermolysin	308-316
LQ**Q**AQV**M**KK	1	50	544.2282	1629.6627	0.23	34	ETD	thermolysin	308-316
VAIMTLKPGTMSPEAFLQ**Q**AQVMK	0	33	1054.1474	3159.4204	-1.31	21	HCD	trypsin	292-315
VAIMTLKPGT**M**SPEAFLQ**Q**AQVMK	1	22	1059.48	3175.4182	-0.4	19	HCD	trypsin	292-315
VAI**M**TLKPGTMSPEAFLQ**Q**AQV**M**K	2	29	1064.8114	3191.4124	-0.62	22	HCD	trypsin	292-315
VAIMTLKPGTMSPEAFLQ**Q**AQVMKK	0	26	1096.8463	3287.5171	-0.73	21	HCD	trypsin	292-316
VAI**M**TLKPGTMSPEAFLQ**Q**AQVMKK	1	21	1102.1786	3303.514	-0.13	20	HCD	trypsin	292-316

VAIMTLKPGTMSPEAFLQ**Q**AQVMK	0	96	790.8646	3159.4295	1.56	115	ETD	trypsin	292-315
VAI**M**TLKPGTMSPEAFLQ**Q**AQVMK	1	60	794.8641	3175.4273	2.49	36	ETD	trypsin	292-315
VAI**M**TLKPGTMSPEAFLQ**Q**AQVMKK	1	32	826.8859	3303.5145	0.032	29	ETD	trypsin	292-315
AIMTLKPGTMSPEAFLQ**Q**A	0	50	859.0386	2574.0939	0.099	31	HCD	elastase	293-311
TLKPGTMSPEAFLQ**Q**A	0	84	1130.4729	2258.9312	-0.34	35	HCD	elastase	296-311
LKPGTMSPEAFLQ**Q**AQVM	0	76	839.692	2516.0541	0.93	33	HCD	elastase	297-314
KPGTMSPEAFLQ**Q**A	0	91	682.6078	2044.8016	0.66	29	HCD	elastase	298-311
KPGTMSPEAFLQ**Q**AQVM	0	40	1202.4913	2402.968	0.13	20	HCD	elastase	298-314
KPGTMSPEAFLQ**Q**AQVMKKLRHEKLV	0	42	707.9456	3534.6917	-0.025	32	ETD	elastase	298-323
LKPGTMSPEAFLQ**Q**AQ	0	45	762.9872	2285.9398	-1.37	32	HCD	thermolysin	297-312
LQ**Q**AQVMKK	0	62	538.8951	1613.6634	-2.48	32	ETD	thermolysin	308-316
LQ**Q**AQV**M**KK	1	50	544.2277	1629.6612	-0.65	36	ETD	thermolysin	308-316

Supplementary Table 4: Summary of all ADP-ribosylated peptides identified by database searching with Mascot with a Mascot Score higher than 15. Modified amino acids (methionine oxidation and ADP-ribosylation) are marked in bold and are underscored; the Mascot PTM site probabilities for these PTM positional isomers are given. All peptides match to the same region of Src-K298M/E310Q; only position 308-311 is covered by all overlapping peptides. Two peptides allow unambiguous localization of the ADP-ribosylation site to position 310 (gray lines, Mascot PTM site probability > 90%). The HCD fragment ion spectrum of the ADP-ribosylated peptide with the sequence KPGTMSPEAFLQQA is shown in Figure 4E.

Supplementary references

1. Levine, M. *et al.*, Escherichia coli strains that cause diarrhoea but do not produce heat-labile or heat-stable enterotoxins and are non-invasive. *Lancet.* 1 (8074), 1119-1122 (1978).
2. Garmendia, J. *et al.*, TccP is an enterohaemorrhagic Escherichia coli O157:H7 type III effector protein that couples Tir to the actin-cytoskeleton. *Cell Microbiol* 6 (12), 1167-1183 (2004).
3. Jerse, A.E., Yu, J., Tall, B.D., & Kaper, J.B., A genetic locus of enteropathogenic Escherichia coli necessary for the production of attaching and effacing lesions on tissue culture cells. *Proc Natl Acad Sci U S A* 87 (20), 7839-7843 (1990).
4. Tzipori, S. *et al.*, Role of a 60-megadalton plasmid and Shiga-like toxins in the pathogenesis of infection caused by enterohemorrhagic Escherichia coli O157:H7 in gnotobiotic piglets. *Infect Immun* 55 (12), 3117-3125 (1987).
5. Munera, D. *et al.*, Recruitment and membrane interactions of host cell proteins during attachment of enteropathogenic and enterohaemorrhagic Escherichia coli. *Biochem J* 445 (3), 383-392 (2012).
6. Guan, K.L. & Dixon, J.E., Eukaryotic proteins expressed in Escherichia coli: an improved thrombin cleavage and purification procedure of fusion proteins with glutathione S-transferase. *Anal Biochem* 192 (2), 262-267 (1991).
7. Tollis, S., Dart, A.E., Tzircotis, G., & Endres, R.G., The zipper mechanism in phagocytosis: energetic requirements and variability in phagocytic cup shape. *BMC Syst Biol* 4, 149 (2010).
8. Clements, A. *et al.*, EspG of enteropathogenic and enterohemorrhagic E. coli binds the Golgi matrix protein GM130 and disrupts the Golgi structure and function. *Cell Microbiol* 13 (9), 1429-1439 (2011).
9. Newsome, T.P., Weisswange, I., Frischknecht, F., & Way, M., Abl collaborates with Src family kinases to stimulate actin-based motility of vaccinia virus. *Cell Microbiol* 8 (2), 233-241 (2006).
